# Chemical screening identifies ROCK as a target for recovering mitochondrial function in Hutchinson‐Gilford progeria syndrome

**DOI:** 10.1111/acel.12584

**Published:** 2017-03-19

**Authors:** Hyun Tae Kang, Joon Tae Park, Kobong Choi, Hyo Jei Claudia Choi, Chul Won Jung, Gyu Ree Kim, Young‐Sam Lee, Sang Chul Park

**Affiliations:** ^1^ Well Aging Research CenterSamsung Advanced Institute of TechnologySamsung ElectronicsSuwon‐siKorea; ^2^ Well Aging Research CenterDGISTDaeguKorea; ^3^ Department of New BiologyDGISTDaeguKorea

**Keywords:** Cytochrome c, HGPS, Rac1b, ROCK, ROS, Y‐27632

## Abstract

Hutchinson‐Gilford progeria syndrome (HGPS) constitutes a genetic disease wherein an aging phenotype manifests in childhood. Recent studies indicate that reactive oxygen species (ROS) play important roles in HGPS phenotype progression. Thus, pharmacological reduction in ROS levels has been proposed as a potentially effective treatment for patient with this disorder. In this study, we performed high‐throughput screening to find compounds that could reduce ROS levels in HGPS fibroblasts and identified rho‐associated protein kinase (ROCK) inhibitor (Y‐27632) as an effective agent. To elucidate the underlying mechanism of ROCK in regulating ROS levels, we performed a yeast two‐hybrid screen and discovered that ROCK1 interacts with Rac1b. ROCK activation phosphorylated Rac1b at Ser71 and increased ROS levels by facilitating the interaction between Rac1b and cytochrome c. Conversely, ROCK inactivation with Y‐27632 abolished their interaction, concomitant with ROS reduction. Additionally, ROCK activation resulted in mitochondrial dysfunction, whereas ROCK inactivation with Y‐27632 induced the recovery of mitochondrial function. Furthermore, a reduction in the frequency of abnormal nuclear morphology and DNA double‐strand breaks was observed along with decreased ROS levels. Thus, our study reveals a novel mechanism through which alleviation of the HGPS phenotype is mediated by the recovery of mitochondrial function upon ROCK inactivation.

## Introduction

Hutchinson‐Gilford progeria syndrome (HGPS) represents one of premature aging syndromes wherein patients die at an average age of 12.6 years (Capell *et al*., 2005). The cardinal feature of HGPS is the generation of truncated lamin A protein, which is induced by point mutations in the *LMNA* gene. This causes irregular/enlarged nuclei and nuclear blebbing (McClintock *et al*., 2006). Current research into HGPS is mainly focused on developing drugs that can reverse these nuclear abnormalities. Farnesyl transferase inhibitors (FTIs), originally developed for cancer treatment, have been shown to reverse the abnormal nuclei phenotype in HGPS fibroblasts (Mehta *et al*., 2010). Additionally, clinical trials of FTIs have yielded improvements in weight gain, vascular stiffness, and bone structure in patients with HGPS (Gordon *et al*., 2012). However, treatment with FTIs is associated with harmful side effects including cytotoxicity and centrosome separation defects (Verstraeten *et al*., 2011). Given these findings, the use of FTIs alone for the treatment of patients with HGPS has been questioned; thus, there is a need for more effective drugs that can be used alone or in combination with FTIs (Liu *et al*., 2006).

Mitochondria generate reactive oxygen species (ROS) as by‐products of the inefficient electron transfer across the electron transport chain (ETC; Quinlan *et al*., 2013). Moderate levels of ROS initiate signaling pathways involved in cell proliferation and differentiation (Devasagayam *et al*., 2004), whereas excessive levels of ROS cause oxidative stress, which in turn induces apoptosis and/or cellular senescence (Gorrini *et al*., 2013). Notably, HGPS fibroblasts generate higher levels of ROS than normal fibroblasts (Richards *et al*., 2011). Furthermore, the basal level of antioxidant enzymes, which defend cells against ROS‐induced damage, is diminished in HGPS fibroblasts as well (Yan *et al*., 1999). Excessive ROS levels constitute the primary cause of oxidative damage in DNA and result in an accumulation of DNA double‐strand breaks (DSBs; Green *et al*., 2011). This causes poor growth and early onset of the senescent phenotype (Chen *et al*., 2007). Thus, therapeutic strategies that reduce ROS levels might prove beneficial to patients with HGPS (Richards *et al*., 2011; Zhavoronkov *et al*., 2012). The value of this approach has been strengthened by results showing that treatment of HGPS fibroblasts with a ROS scavenger (N‐acetylcysteine; NAC) reduces DNA DSBs and improves cell growth rates (Richards *et al*., 2011).

In this study, we aimed to identify compounds that can reduce ROS levels using high‐throughput screening (HTS). From this analysis, we selected a ROCK inhibitor (Y‐27632) as an effective agent. As the role of ROCK in the regulation of ROS remains elusive, we performed a yeast two‐hybrid screen and identified a novel interaction between ROCK1 and Rac1b. Here, we demonstrated that ROCK could regulate ROS generation by modulating the interaction between Rac1b and cytochrome c.

## Results

### HTS for compounds that reduce ROS levels in senescent HGPS fibroblasts

**Figure 1:**
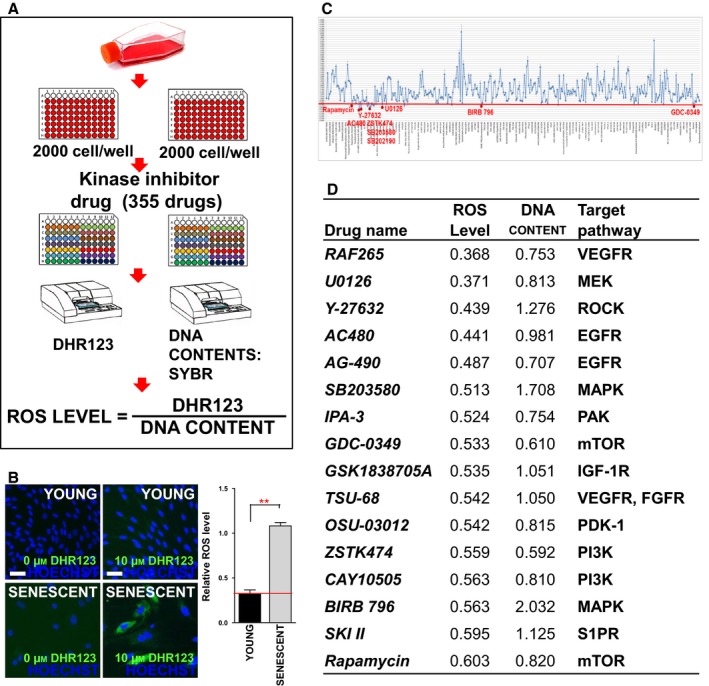
HTS for compounds that reduce ROS levels in senescent HGPS fibroblasts. (A) Experimental procedure for assessing ROS levels using DHR123 values normalized to DNA content. (B) DHR123 staining (green) in young and senescent HGPS. Nuclei were counterstained with Hoechst 33342 (blue; Scale bar = 10 μm). Relative ROS levels in young and senescent HGPS. (**P <* *0.01, one‐way ANOVA) Means ± SD, *N* = 3. (C) Statistically significant changes in ROS reduction were defined as less than 0.61‐fold compared to DMSO controls (red line). (D) Candidate compounds identified based on ROS levels and DNA content. Means, *N* = 6.

The present screening strategy consisted of two different methods to evaluate the capacity of restoration in senescent HGPS fibroblasts: (i) reducing ROS levels and (ii) increasing cell numbers. For the primary screen, a DHR123 fluorescence‐based method for measuring mitochondrial hydroxyl radicals/hydrogen peroxide and a DNA content‐based method for measuring cell numbers were combined. ROS levels were determined by normalizing DHR123 values with DNA content (Fig. 1A). Through use of this system, a significant difference in ROS levels was observed between young and senescent HGPS fibroblasts (Fig. 1B). A library containing 355 kinase inhibitors was added to senescent HGPS fibroblasts, and their effect on ROS levels was determined on day 16 (Fig. 1C and Table S1, Supporting information). The inhibitors that led to <0.61‐fold compared to DMSO controls were considered potential hits and sixteen compounds were identified as candidate drugs (Fig. 1C,D). The demonstration of proliferation‐inducing effect was considered to select the best candidate drug, because cell cycle arrest represents one of the hallmarks of senescence (Dechat *et al*., 2007). However, even though RAF265 and U0126 were ranked first and second, they did not induce cell proliferation (Fig. 1D; DNA contents 0.753 and 0.813, respectively). Conversely, Y‐27632 triggered cell proliferation as well as reduced ROS levels (Fig. 1D; DNA content 1.276). Based on these data, Y‐27632 (ROCK inhibitor) was selected as a candidate drug that restores senescent HGPS fibroblasts by reducing ROS levels and inducing cell proliferation.

### ROCK as a potential target for reducing ROS levels

**Figure 2:**
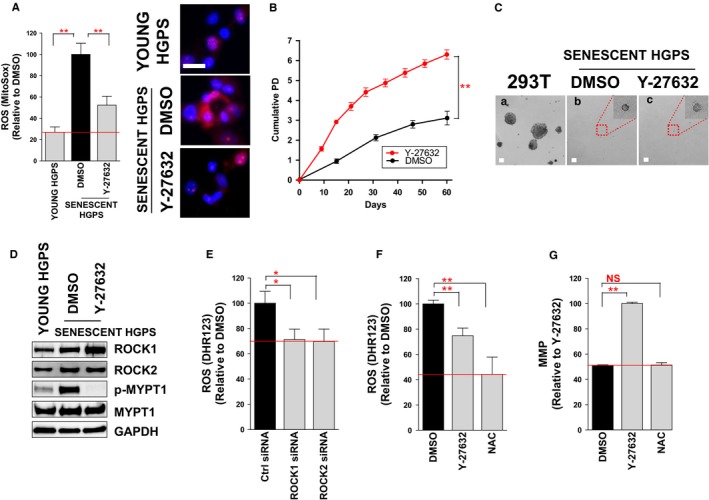
ROCK as a potential target for reducing ROS levels. (A) Flow cytometric analysis of mitochondrial ROS levels using MitoSOX (**P <* *0.01, one‐way ANOVA). Mean ± SD, *N* = 3. MitoSOX staining (magenta) in young and senescent HGPS. Nuclei were counterstained with Hoechst 33342 (blue; Scale bar = 10 μm). (B). Effects of Y‐27632 treatment on cumulative population doublings (**P <* *0.01, one‐way ANOVA). (C) Soft agar assay (scale bar = 20 μm). (D) Specificity of Y‐27632 as a ROCK inhibitor. (E and F) Flow cytometric analysis of mitochondrial ROS levels using DHR123 (**P <* *0.01, *P <* *0.05 one‐way ANOVA). Means ± SD, *N* = 3. (G) Flow cytometric analysis of MMP using JC‐1 (NS: not significant, **P <* *0.01, one‐way ANOVA). Means ± SD, *N* = 3.

To further confirm the ROS‐reducing effect of Y‐27632, ROS levels were measured using a different dye, MitoSox, to detect mitochondrial superoxide anions. Y‐27632 treatment decreased ROS levels similar to that observed in HTS (Fig. 2A). Next, to confirm the proliferation‐inducing effect of Y‐27632, cumulative population doubling (CPD) and p16INK4a (p16) expression level were measured. Upon Y‐27632 treatment, CPD was increased concomitant with decreased p16 expression (Fig. 2B and Fig. S1, Supporting information). Because cells were triggered to grow linearly upon Y‐27632 treatment, a soft agar assay was performed to exclude the possibility that cells may undergo cancerous transformation. Although HEK 293T cells (CRL‐11268; ATCC) formed colonies as an indicator of transformation, Y‐27632 treatment did not induce colony formation in senescent HGPS fibroblasts (Fig. 2C).

Next, the specificity of Y‐27632 as a ROCK inhibitor was tested. Y‐27632 treatment did not influence the expression levels of ROCK1 or ROCK2, whereas it markedly decreased the phosphorylation level of a ROCK downstream signal (p‐MYPT1; Fig. 2D). This result implies that ROCK activation is specifically attenuated by Y‐27632. We then examined whether the inhibition of *ROCK* isoforms by short interfering (si) RNA treatment would have a similar effect as Y‐27632 treatment. ROS levels were reduced by knockdown of either *ROCK1* or *ROCK2* (Fig. 2E). To further validate that the ROS‐reducing effect by Y‐27632 arises from an on‐target effect of this drug, we examined whether Y‐27632 treatment could further decrease ROS levels in ROCK1‐ or ROCK2‐deficient cells. ROS levels were decreased in control siRNA‐treated cells upon Y‐27632 treatment (Fig. S2A–C, Supporting information). Similarly, cells lacking ROCK1 or ROCK2 exhibited the reduction in ROS levels (Fig. S2A–C, Supporting information). However, these cells failed to further decrease ROS levels after Y‐27632 treatment (Fig. S2A–C, Supporting information). These results imply that the ROS‐reducing effect of Y‐27632 is achieved by regulating the activities of ROCK isoforms.

We then compared the ROS‐reducing effect of Y‐27632 with that of a widely used ROS scavenger, N‐acetylcysteine (NAC). As expected, ROS levels were significantly reduced following NAC treatment (Fig. 2F). Oxidative damage induced by ROS has a detrimental effect on mitochondrial function including mitochondrial membrane potential (MMP; Yen & Klionsky, 2008). Thus, we examined whether Y‐27632 has an additional role in regulating mitochondrial function. MMP was significantly increased by Y‐27632 treatment, but was not influenced by NAC treatment (Fig. 2G and Fig. S3, Supporting information). This result implies that the effect of Y‐27632 on the recovery of MMP does not arise from a simple ROS‐reducing effect.

### ROCK1 interacts with Rac1b *in vitro* and *in vivo*

**Figure 3:**
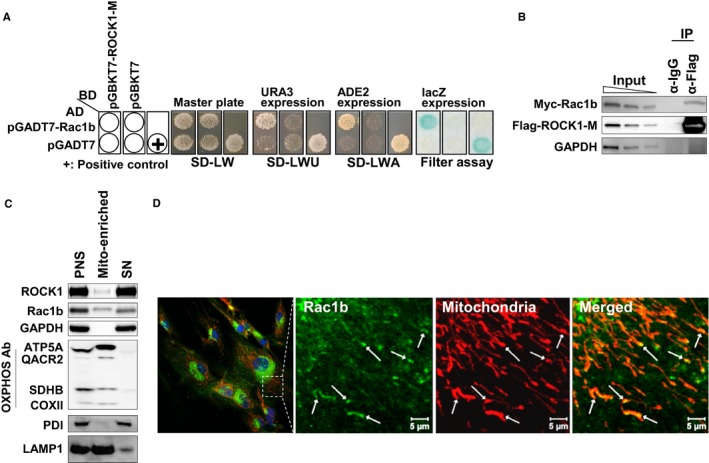
ROCK1 interacts with Rac1b *in vitro* and *in vivo*. (A) The AH109 yeast strain was transformed with either the GAL4‐BD fusion plasmid pGBKT7 or pGBKT7‐ROCK1‐M. The pGADT7‐Rac1b clone was selected for further study. (B) Co‐immunoprecipitation of Flag‐ROCK1‐M with Myc‐Rac1b. (C) Localization of ROCK1 and Rac1b (Postnuclear supernatant (PNS), mitochondrial fraction (Mito‐enriched: pellet), or cytosol (SN: supernatant)). (D) Confocal imaging of Rac1b (green) and mitochondria (red; Scale bar = 5 μm).

ROCK is known to regulate the shape and movement of cells by acting on the cytoskeleton (Amano *et al*., 2010). A recent study has shown that ROCK regulates mitochondrial function through modulating mitochondrial dynamics (Wang *et al*., 2012). However, the underlying mechanism of ROCK in regulating mitochondrial function remains elusive. ROCK has a range of substrates and interaction partners and plays pivotal roles in numerous cellular functions through their individual regulation (Amano *et al*., 2010). The identification of novel interaction partners for ROCK may help to reveal its role in mitochondrial function; thus, we performed a yeast two‐hybrid screen. As baits, the Rho‐binding domains in ROCK1 and ROCK2 were used: ROCK1‐M (residues 945–1113, 169 aa) and ROCK2‐M (residues 976–1131, 156 aa). A single positive clone from ROCK1‐M and three positive clones from ROCK2‐M were identified after screening 2.5 × 10^6^ clones with three reporter genes (Table S2, Supporting information). Among these, we focused on the interaction between ROCK1‐M and Rac1b because Rac1b is known to induce mitochondrial dysfunction through stimulating mitochondrial ROS generation (Radisky *et al*., 2005; Fig. 3A). To confirm the interaction between ROCK1‐M and Rac1b, HEK 293T cells were transiently transfected with Flag‐ROCK1‐M and Myc‐Rac1b. Flag‐ROCK1‐M co‐immunoprecipitated with Myc‐Rac1b, confirming their direct interaction (Fig. 3B).

Rac1b, an alternatively spliced form of Rac1, comprises a small GTP binding protein (Radisky *et al*., 2005). It is characterized by an in‐frame insertion of 57 nucleotides (residues 229–285) into *Rac1* mRNA. Rac1 can translocate into mitochondria via a conserved cysteine motif that serves as a potential mitochondrial localization signal (Osborn‐Heaford *et al*., 2012). As Rac1b also possesses this motif, we examined the localization of Rac1b to the mitochondria by subcellular fractionation and immunofluorescence. Postnuclear supernatant (PNS) was submitted to high‐speed centrifugation to separate the mitochondrial fraction (Mito‐enriched fraction: pellet) from the cytosol (SN: supernatant). Oxidative phosphorylation (oxphos) complex proteins were highly abundant in the mito‐enriched fraction and Rac1b was observed in this fraction as well (Fig. 3C).

To verify the mitochondrial purity in mito‐enriched fraction, we did immunoblot against ER (Protein disulfide isomerase; PDI) and lysosomal marker (lysosomal‐associated membrane protein 1; LAMP1). As expected, the ER marker (PDI) was highly enriched in PNS and SN fraction but nearly absent in the mito‐enriched fraction. Rac1b has the same CAAX motif at the C‐terminus as Rac1 (Radisky *et al*., 2005). This motif is required for membrane targeting and is processed by RCE1 peptidase residing at the surface of the ER (Gao *et al*., 2009). As the level of ER contamination was very low in mito‐enriched fraction, the chance of ROCK1–Rac1b interaction on ER membrane would be very low. Lysosomal marker (LAMP1) was abundant in PNS and mito‐fraction. The lysosomal contamination in mito‐fraction is considered to come from our mitochondria fractionation method because it was based on the crude isolation to purify the heavy weight particles including mitochondria (He *et al*., 2011). However, confocal microscopy analysis confirmed the localization of Rac1b to the mitochondria (Fig. 3D; white arrow). Taken together, even though mito‐enriched fraction is contaminated with lysosomal fraction, our results suggest that ROCK1–Rac1b interaction occurred in the mitochondria.

### ROCK phosphorylates Rac1b at Ser 71

**Figure 4:**
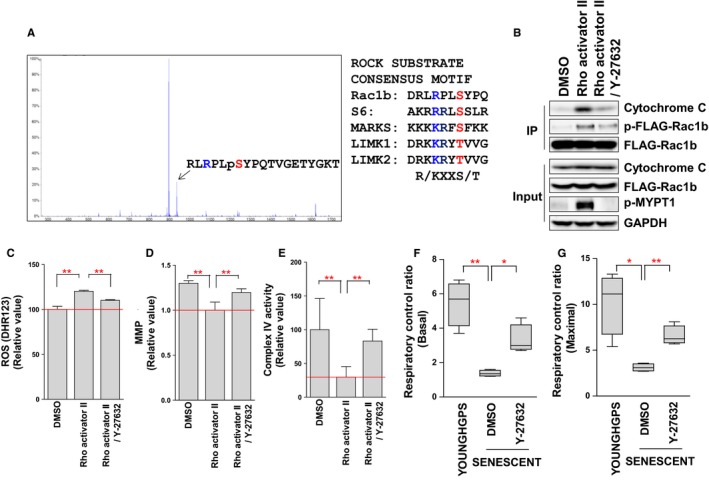
ROCK regulates mitochondrial ROS generation by modulating the interaction between Rac1b and cytochrome c. (A) Identification of the Rac1b phosphorylation site by MALDI‐TOF mass spectrometry. Comparison of the phosphorylation sequence in Rac1b with those of other ROCK substrates. (B) RhoA/ROCK activation increased the interaction between Rac1b and cytochrome c through the phosphorylation of Rac1b at Ser71. (C) Flow cytometric analysis of mitochondrial ROS levels using DHR123 (**P <* *0.01, one‐way ANOVA). Means ± SD, *N* = 3. (D) Flow cytometric analysis of MMP using JC‐1 (**P <* *0.01, one‐way ANOVA). Means ± SD, *N* = 3. (E) Assessment of COX activity by measuring oxygen consumption. (**P <* *0.01, one‐way ANOVA). Means ± SD, *N* = 3. (F and G) Measurement of basal and maximal RCR (**P <* *0.01, *P <* *0.05, one‐way ANOVA). Means ± SD, *N* = 3.

Protein phosphorylation regulates protein–protein interactions as well as the function of target proteins (Tudor *et al*., 2015). Because we identified that ROCK1 interacted with Rac1b, we hypothesized that ROCK1 would also modulate Rac1b activity via phosphorylation. To identify the phosphorylation site in Rac1b, MALDI‐TOF mass spectrometry was performed. HEK 293T cells were transiently transfected with Flag‐Rac1b. To induce RhoA/ROCK activation, HEK 293T cells were treated with Rho Activator II, which activates RhoA by deamidating glutamine‐63 (Riento & Ridley, 2003). We identified an abundant deprotonated molecule of *m/z* 946.00 corresponding to the amino acid sequence RLRPLpSYPQTVGETYGKT (Fig. 4A), which contained the phosphorylation consensus motif by ROCK: R/KXXS/T (R, arginine; K, lysine; X, any amino acid; S, serine; T, threonine; Sumi *et al*., 2001). Furthermore, the sequence flanking the identified phosphorylation site in Rac1b was similar to consensus motif of other ROCK substrates such as S6, MARKS, LIMK1, and LIMK2 (Fig. 4A). We then examined whether ROCK could phosphorylate Rac1b *in vivo*. HEK 293T cells that had been transfected with Flag‐Rac1b were treated with Rho Activator II or co‐treated with Rho Activator II/Y‐27632. Phosphorylation of the immunoprecipitated Rac1b was recognized using a phospho‐serine/threonine antibody. Notably, its immunoreactivity was increased by treatment with Rho Activator II but was decreased by co‐treatment with Y‐27632 (Fig. 4B).

### ROCK regulates mitochondrial ROS generation by modulating the interaction between Rac1b and cytochrome c

Rac1 is a member of the Rho GTPase family and regulates multiple cellular processes including cytoskeleton reorganization and motility (Radisky *et al*., 2005). A recent study shows that Rac1 regulates mitochondrial ROS generation via its interaction with cytochrome c (Osborn‐Heaford *et al*., 2012). As we had observed that mitochondrial ROS levels were reduced upon Y‐27632 treatment, we hypothesized that (i) Rac1b would interact with cytochrome c, and (ii) ROCK would regulate ROS levels by modulating the interaction between Rac1b and cytochrome c. We found that Rac1b interacted with cytochrome c and that its interaction was increased by RhoA/ROCK activation but was decreased by co‐treatment with Y‐27632 (Fig. 4B). The increased association of Rac1b with cytochrome c was expected to increase ROS levels. Indeed, we observed that ROS levels were increased by RhoA/ROCK activation (Fig. 4C and Fig. S4A, Supporting information). Notably, ROS levels were decreased by co‐treatment with Y‐27632 (Fig. 4C). We then examined whether ROCK also regulates MMP. Although RhoA/ROCK activation decreased MMP, co‐treatment with Y‐27632 restored MMP (Fig. 4D and Fig. S4B, Supporting information). These results imply that ROCK regulates mitochondrial function through modulating the interaction between Rac1b and cytochrome c.

To further verify whether the ROCK–Rac1b–cytochrome c axis plays an important role in regulating ROS levels, we examined whether Y‐27632 treatment could decrease ROS generation in Rac1b*‐*deficient cells. ROS levels were decreased in control siRNA‐treated cells after Y‐27632 treatment (Fig. S2D, Supporting information). Similarly, cells lacking Rac1b exhibited the reduction in ROS levels. However, these cells failed to further decrease ROS generation following Y‐27632 treatment (Fig. S2D, Supporting information). This result implies that the ROS‐reducing effect of Y‐27632 is achieved by regulating the ROCK–Rac1b–cytochrome c axis.

### ROCK regulates mitochondrial function by modulating COX activity

Cytochrome c is a component of the ETC. in mitochondria (Kadenbach *et al*., 2000). The heme group of cytochrome c accepts electrons from complex III and transfers them to complex IV (cytochrome c oxidase; COX; Zorov *et al*., 2014). In turn, reduced COX is efficiently oxidized by O_2_, which drives proton pump activity across the inner mitochondrial membrane (Belevich *et al*., 2006). Emerging evidence suggests that COX dysfunction is invariably associated with increased mitochondrial ROS generation (Srinivasan & Avadhani, 2012). Thus, we examined whether COX activity is regulated by the ROCK–Rac1b–cytochrome c axis. To assess COX activity, oxygen consumption in COX was measured using permeabilized cells with intact mitochondria (Salabei *et al*., 2014). Notably, RhoA/ROCK activation decreased COX activity whereas co‐treatment with Y‐27632 restored its activity (Fig. 4E). Thus, ROCK‐induced ROS generation can be explained by the ROCK‐induced deterioration of COX activity.

Deficiency in COX activity leads to a compromised MMP, which in turn decreases oxphos efficiency (Li *et al*., 2006). As we observed that ROCK regulates COX activity, we hypothesized that ROCK also regulates oxphos efficiency. To confirm this, the respiratory control ratio (RCR) was measured as an indicator of mitochondrial respiration (Brand Martin & Nicholls David, 2011). Senescent HGPS fibroblasts demonstrated lower basal (without the addition of FCCP) and maximal (with the addition of FCCP) RCR than young HGPS fibroblasts, implying that senescent HGPS fibroblasts exhibit a defective mitochondrial respiration (Fig. 4F,G). However, Y‐27632 treatment recovered RCR to a level similar to that of young HGPS fibroblasts (Fig. 4F,G). Taken together, these results suggest that ROCK inactivation by Y‐27632 induces the recovery of mitochondrial function by improving the efficiency of oxphos.

### ROCK regulates mitochondrial function by inducing metabolic reprogramming

**Figure 5:**
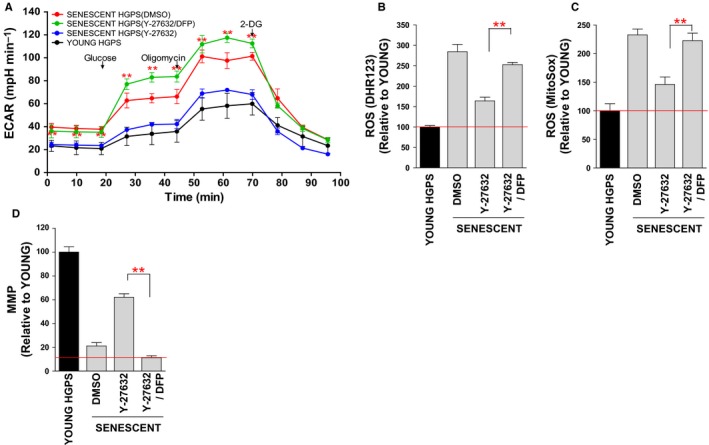
ROCK regulates mitochondrial function by inducing metabolic reprogramming. (A) Measurement of ECAR (black line: young HGPS fibroblasts, blue line: Y‐27632‐treated senescent HGPS fibroblasts, green line: Y‐27632/DFP‐treated senescent HGPS fibroblasts, and red line: DMSO‐treated senescent HGPS fibroblasts; **P <* *0.01, one‐way ANOVA). Means ± SD, *N* = 3. (B and C) Flow cytometric analysis of mitochondrial ROS levels using DHR123 (B) and MitoSOX (C) (**P <* *0.01, one‐way ANOVA). Means ± SD, *N* = 3. (D) Flow cytometric analysis of MMP using JC‐1 (**P <* *0.01, one‐way ANOVA). Means ± SD, *N* = 3.

HGPS fibroblasts exhibit profound metabolic alterations, suggestive of metabolic reprogramming from oxphos to glycolysis (Rivera‐Torres *et al*., 2013). As we had observed that ROCK regulates oxphos efficiency, we hypothesized that ROCK could regulate metabolic reprogramming as well. Therefore, we measured the extracellular acidification rate (ECAR) as an indicator of glycolytic flux. The observed ECAR of senescent HGPS fibroblasts was higher than that of young HGPS fibroblasts, implying that senescent HGPS fibroblasts show increased dependency on glycolysis to meet their energy demands (Fig. 5A; black line vs. red line). However, ROCK inactivation with Y‐27632 reduced ECAR to that of young HGPS fibroblasts, implying that ROCK inactivation induces metabolic reprogramming from glycolysis to oxphos (Fig. 5A; blue line).

To determine whether metabolic reprogramming plays an important role in the recovery of mitochondrial function, we co‐treated senescent HGPS fibroblasts with an aconitase inhibitor, deferiprone (DFP), that acts to shift the metabolism from oxphos to glycolysis (Goncalves *et al*., 2008). DFP treatment hampered the Y‐27632‐induced metabolic reprogramming by increasing ECAR (Fig. 5A; green line). Furthermore, DFP treatment hindered the Y‐27632‐induced recovery of mitochondrial function, accompanied by increased ROS levels and decreased MMP (Fig. 5B–D). These data suggest that metabolic reprogramming driven by Y‐27632 is important for the recovery of mitochondrial function.

### Reduced frequency of abnormal nuclear morphology and DNA DSBs upon Y‐27632 treatment

**Figure 6:**
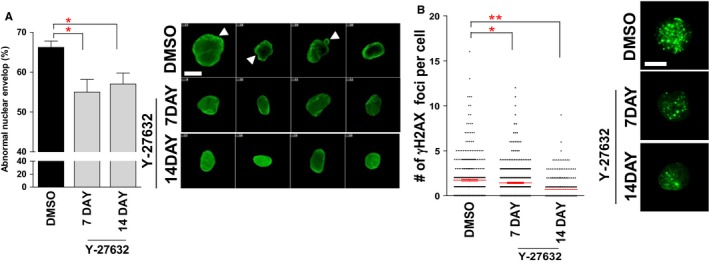
Reduced frequency of abnormal nuclear morphology and DNA DSBs upon Y‐27632 treatment. (A) Measurement of abnormal nuclear structures (green: lamin A/C, arrow head: abnormal nuclear structure, scale bar = 5 μm; *P <* *0.05, one‐way ANOVA). Means ± SD, *N* = 100. (B) Measurement of γH2AX foci (green: γH2AX, blue: DAPI, scale bar = 5 μm; **P <* *0.01, *P <* *0.05, one‐way ANOVA). Means ± SD, *N* = 400.

Oxidative stress is suggested to be one of the primary cause of abnormal nuclear morphology owing to the oxidation of conserved cysteine residues in lamin A (Pekovic *et al*., 2011). As we observed that ROS levels were reduced by Y‐27632 treatment, we conjectured that this effect may decrease the frequency of abnormal nuclear morphology. Consistent with this hypothesis, Y‐27632 treatment markedly reduced the frequency of misshapen nuclei in HGPS cells (Fig. 6A). In addition, excessive ROS is known to cause the formation of DNA DSBs during senescence (Green *et al*., 2011). Thus, we evaluated the prevalence of DNA DSBs. Y‐27632 treatment decreased DNA DSBs’ frequency, as indicated by the decreased number of γH2AX foci (Fig. 6B). These results imply that the ROS reduction driven by Y‐27632 may ameliorate the abnormal nuclear morphology accompanied by a reduction in DNA DSBs.

To further validate that the amelioration of misshapen nuclei by Y‐27632 arises from an on‐target effect of this drug, we examined Y‐27632 effects on nuclear morphology in senescent HGPS fibroblasts lacking ROCK1, ROCK2, or Rac1b. The frequency of misshapen nuclei was decreased in control siRNA‐treated cells after Y‐27632 treatment (Fig. S2E, Supporting information). Moreover, it was decreased in cells lacking ROCK1, ROCK2, or Rac1b (Fig. S2E, Supporting information). However, these cells failed to further decrease abnormal nuclear morphology even after Y‐27632 treatment (Fig. S2E, Supporting information). Taken together, these data suggest that the amelioration of misshapen nuclei by Y‐27632 may be achieved by regulating ROCK–Rac1b–cytochrome c axis.

## Discussion

Oxidative stress is closely linked to the control of aging and age‐related diseases. ROS levels are 5‐fold higher in HGPS fibroblasts than in normal fibroblasts (Richards *et al*., 2011). The significance of reducing ROS levels in HGPS is supported by the findings that treatment of HGPS fibroblasts with a ROS scavenger (NAC) reduces the levels of DNA damages (Richards *et al*., 2011; Kubben *et al*., 2016) and even improves their proliferation rates to some extent (Richards *et al*., 2011). However, the underlying mechanism of increased ROS generation in HGPS is poorly understood. A previous study has shown that p66Shc triggers mitochondrial ROS generation by oxidizing cytochrome c (Giorgio *et al*., 2005). Similarly, Rac1 produces mitochondrial ROS through directly intercepting electrons from cytochrome c as does p66Shc (Osborn‐Heaford *et al*., 2012). In the current study, we uncovered a novel mechanism in which ROCK regulates mitochondrial ROS generation by modulating the interaction between Rac1b and cytochrome c. RhoA/ROCK activation induced the phosphorylation of Rac1b at Ser 71 and facilitated the interaction between Rac1b and cytochrome c. This interaction could induce the partial reduction in oxygen by intercepting electrons from cytochrome c in a similar manner as p66Shc and Rac1. On the contrary, ROCK inactivation with Y‐27632 hampered the interaction between Rac1b and cytochrome c. Taken together, the results from our study reveal the novel roles of ROCK as a binding partner for Rac1b and a regulator of mitochondrial ROS generation.

Mitochondrial dysfunction is considered to be both a target of aging and a contributor to it (Bratic & Larsson, 2013). Furthermore, alteration of mitochondrial function in HGPS may contribute to premature organ decline (Rivera‐Torres *et al*., 2013); however, the means by which to delay or prevent this alternation are poorly understood. The primary function of mitochondria is the production of ATP to meet cellular energy demand. To this end, mitochondrial respiratory complex I, complex III, and complex IV (COX) generate MMP by transferring protons from the matrix to the intermembrane space. MMP is diminished primarily by ROS‐induced mitochondrial damage (James *et al*., 2015). Although the involvement of ROS in mitochondrial depolarization has attracted much attention (Bernardi *et al*., 2006), a consensus on its role in MMP has not been achieved. A recent study suggests that deterioration in COX activity leads to mitochondrial dysfunction through decreasing MMP (Li *et al*., 2006). Here, we observed that RhoA/ROCK activation decreased MMP accompanied by a deterioration in COX activity, whereas Y‐27632 treatment recovered MMP along with increased COX activity. Y‐27632‐induced‐MMP improvement may be plausibly explained by increased COX activity upon ROCK inactivation with Y‐27632. Our findings therefore suggest a novel mechanism in which ROCK regulates MMP by modulating COX activity.

In HGPS fibroblasts, severe mitochondrial dysfunction has been observed along with a marked decrease in COX activity and a significant increase in glycolytic dependency (Rivera‐Torres *et al*., 2013). Support for this finding is evident from the observation that HGPS fibroblasts show metabolic alterations that suggest a metabolic switch from oxphos to glycolysis (Aliper *et al*., 2015). To our knowledge, the present study provides the first demonstration that Y‐27632 treatment restores mitochondrial respiration, which is altered in senescent HGPS fibroblasts, through increasing the efficiency of oxphos. Notably, we found that this improvement is accompanied by diminished dependency on glycolysis as an energy source. Extending the relevance of these findings, the forced metabolic shift from oxphos to glycolysis by an aconitase inhibitor blocked the Y‐27632‐induced recovery of mitochondrial function, implying that metabolic reprogramming is necessary for the functional recovery of mitochondria. Taken together, the findings from our study suggest that ROCK inactivation with Y‐27632 facilitates the functional recovery of mitochondria by inducing metabolic reprogramming.

A common feature of HGPS is the presence of single mutations in exon 11 of the *LMNA* gene. These mutations generate a truncated protein, which is defective in the release of a farnesyl group. The retention of the farnesyl group in progerin induces defect in nuclear morphology (McClintock *et al*., 2006). Thus, the development of specific pharmacological drugs targeting HGPS has focused on reversing nuclear abnormality. FTIs were recently found to significantly improve the symptoms of HGPS through preventing nuclear blebbing (Capell *et al*., 2005). However, FTIs do not reduce the DNA damage load (Gordon *et al*., 2014) and they exhibit several adverse effects including centrosome separation defects, nuclear dysmorphy, and cytotoxicity (Verstraeten *et al*., 2011); thus, their use as a potential treatment option for HGPS has been questioned. In the present study, we observed a reduction in the frequency of abnormal nuclear morphology along with a reduction in DNA DSBs. Furthermore, we observed that ROS levels, which are known to induce abnormal nuclear morphology through the oxidation of conserved cysteine residues in lamin A (Pekovic *et al*., 2011), were decreased upon Y‐27632 treatment. Based on these findings, we conclude that ROCK inactivation with Y‐27632 may serve as one of the potential means of ameliorating both abnormal nuclear morphology and DNA DSBs in HGPS. We propose that the use of Y‐27632 alone or in combination with FTIs would be effective for HGPS treatment, although further studies are required to confirm this outcome.

The significance of the ROCK inhibitor in replicative lifespan (RLS) is highlighted by the finding that it extended the RLS of normal keratinocytes (Chapman *et al*., 2010, 2014). Y‐27632 greatly increased the proliferative capacity of keratinocytes without detectable cell crisis (Chapman *et al*., 2010, 2014). In our study, when we added Y‐27632 on senescent HGPS fibroblasts, we observed the increase in the cumulative population doubling (CPD) concomitant with decreased p16 expression. Based on previous studies and our observation, we propose that the addition of Y‐27632 at an earlier PD would likely increase the magnitude of RLS extension; this possibility needs to be elucidated in further studies.

In summary, our findings unravel a mechanism through which the adjustment of ROCK activity improves mitochondrial abnormalities and may pave the way for effective treatment for patient with HGPS (Fig. S5, Supporting information). These studies may also improve our knowledge of the mechanisms leading to mitochondrial dysfunction during aging. Thus, our study suggests that the adjustment of ROCK activity may be clinically applied to control premature aging and aging‐related disease.

## Experimental procedures

### Cell culture

HGPS skin fibroblasts (AG03198 B; Coriell Cell Repositories, Camden, NJ, USA), human diploid fibroblasts (PCS‐201‐010; American Type Culture Collection, Manassas, VA, USA), and HEK 293T cells (CRL‐11268; ATCC) were used in this study. Cells were cultured in Dulbecco's modified Eagle's medium containing 25 mm glucose supplemented with 10% fetal bovine serum, 100 U mL^−1^ penicillin, and 100 μg mL^−1^ streptomycin. Cells were also cultured in ambient air (20% O_2_) supplemented with 5% CO_2_. Confluent cells were split 1:4 and 1:2 during early and late passages, respectively. The culture medium was changed every 4 days. The population doubling level (PDL) was 10 when HGPS skin fibroblasts (AG03198 B; Coriell Cell Repositories) were initially purchased from Coriell Cell Repositories. The approximate PDL of young cells was 14, and the population doubling time was less than 2 days. Cells were considered to be senescent when the population doubling time was over 14 days and the approximate PDL of senescent cells was 26.

### Drug screening

Senescent HGPS cells were seeded in 96‐well plates at a density of 2000 cells/well. We prepared two 96‐well replicates: one for measuring the DHR123 value and the other for measuring cell proliferation. Components of the Kinase Inhibitor Library (L1200; Selleck Chem, Houston, TX, USA) were diluted to a final concentration of 5 μm and added to wells every 4 days until measurements were made at 16 days. For ROS detection, cells were incubated with 10 μm DHR123 (D632; Life Technologies, Carlsbad, CA, USA) for 30 min at 37 °C, washed twice with phosphate‐buffered saline (PBS), and analyzed using an Infinite 200 PRO fluorescence microplate reader (Tecan, Männedorf, Switzerland). Cell number was evaluated by measuring the fluorescence intensity of SYBR Green I nucleic acid gel stain (S‐7567; Molecular Probes, Eugene, OR, USA) on a fluorescence microplate reader. ROS levels were calculated by normalizing DHR123 values with DNA content. The means ± SD from six replicates were determined for each experimental group.

### Measurement of ROS and MMP

For quantitation of mitochondrial ROS, cells were incubated with 30 μm DHR123 (D632; Life Technologies) and 5 μm MitoSOX (M36008; Life Technologies) for 30 min at 37°C, washed with PBS, trypsinized, collected in PBS, and analyzed on a LSRFortessa instrument (Becton Dickinson, Franklin Lakes, NJ, USA). For measurement of the mitochondrial membrane potential, cells were incubated with 0.6 μg ml^−1^ JC‐1 (Invitrogen, Carlsbad, CA, USA) for 30 min at 37°C and prepared for flow cytometry analysis as previously described (Kang & Hwang, 2009).

### Western blot analysis

Protein lysates were prepared by resuspending cell pellets in Laemmli sample buffer containing 5% β‐mercaptoethanol. Protein lysates were then separated by electrophoresis on 4–12% gradient Tris‐glycine gels and transferred onto polyvinylidene difluoride membranes using a semidry apparatus (Bio‐Rad, Hercules, CA, USA). The membrane was blocked with 5% nonfat dry milk in TBST (20 mm Tris–HCl, 0.5 m NaCl, and 0.1% Tween‐20) and incubated with primary antibodies at room temperature for 3 h, followed by washing with TBST. Subsequently, the membrane was incubated with horseradish peroxidase (HRP)‐conjugated secondary antibodies and detected with enhanced chemiluminescence solution (32106; Thermo Scientific, Waltham, MA, USA). Primary antibodies used in this study included mouse anti‐ROCK1 (SC‐17794; 1:1000 dilution; Santa Cruz Biotechnology, Santa Cruz, CA, USA), rabbit anti‐ROCK2 (SC‐5561; 1:1000 dilution; Santa Cruz Biotechnology), rabbit anti‐phospho‐MYPT1 (4563s; 1:1000 dilution; Cell Signaling Technology, Beverly, MA, USA), rabbit anti‐MYPT1 (2634s; 1:1000 dilution; Cell Signaling Technology), mouse anti‐GAPDH (G041; 1:1000 dilution; ABM, Richmond, BC, Canada), rabbit anti‐c‐Myc (C3956‐.2MG; 1:1000 dilution; Sigma, St. Louis, MO, USA), mouse anti‐Flag (A8592; 1:1000 dilution; Sigma), rabbit anti‐Rac1b (09‐271; 1:1000 dilution; Millipore, Darmstadt, Germany), mouse anti‐OXPHOS (MS601; 1:1000 dilution; MitoSciences, Eugene, OR, USA), mouse anti‐PDI antibody (ab2792; 1:1000 dilution; Abcam, Cambridge, UK), mouse anti‐LAMP1 (SC‐20011; 1:1000 dilution; Santa Cruz Biotechnology), mouse anti‐cytochrome c (556433; 1:1000 dilution; BD Biosciences, Franklin Lakes, NJ, USA), and mouse anti‐phospho‐(Ser/Thr; 612548; 1:1000 dilution; BD Biosciences). The secondary antibodies used in this study included HRP‐conjugated anti‐rabbit IgG (sc‐2004; 1:4000 dilution; Santa Cruz Biotechnology) and HRP‐conjugated anti‐mouse IgG (sc‐2302; 1:4000 dilution; Santa Cruz Biotechnology).

### Statistical analyses

Statistical analyses were performed using a standard statistical software package (SigmaPlot 12.5; Systat Software, San Jose, CA, USA). One‐way analysis of variance (ANOVA) was used to determine whether differences were significant.

## Funding

This research was supported by Samsung Advanced Institute of Technology, and the DGIST R&D Program of the Ministry of Science, ICT and Technology of KOREA (20160165 and 20160172).

## Author contributions

JTP, HTK, KBC, and SCP conceived of and designed the experiments. JTP, HTK, KBC, HJC, CWJ, GRK, and YSL performed the experiments. JTP supervised all of experiments. JTP, HTK, and KBC analyzed the data. JTP, HTK, YSL, and SCP wrote and edited the manuscript.

## Competing interests

The authors declare no competing financial interests.

## Supporting information


**Fig. S1** Effect of Y‐27632 on p16 expression.

**Fig. S2** Effect of Y‐27632 on *ROCK1, ROCK2, or Rac1b* deficient HGPS fibroblasts.

**Fig. S3** Effect of Y‐27632 on the recovery of MMP.

**Fig. S4** ROCK regulates mitochondrial function by modulating ROS levels and MMP in HEK 293T cells.

**Fig. S5** Proposed mechanism accounting for the regulation of mitochondrial function via the ROCK‐Rac1b‐cytochrome c axis.

**Fig. S6** Effect of Y‐27632 on mitochondrial mass.

**Table S1** Detailed list of ROS levels in high‐throughput screening.

**Table S2** Detailed list of positive clones from yeast two‐hybrid screening.

**Appendix S1** Experimental procedures.
