# Supplementary figures and images for: Chemical screening identifies ROCK as a target for recovering mitochondrial function in Hutchinson‐Gilford progeria syndrome
Source: Aging Cell. 2017 Mar 19;16(3):541–50. doi: 10.1111/acel.12584 (PMC5418208; doi:10.1111/acel.12584)

Supplementary Figure 1

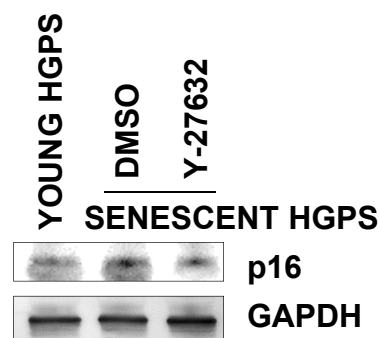

Supplement: Supplementary file 1 — Fig. S1 Effect of Y‐27632 on p16 expression. [file ACEL-16-541-s001.pdf]

## Supplementary Figure 2

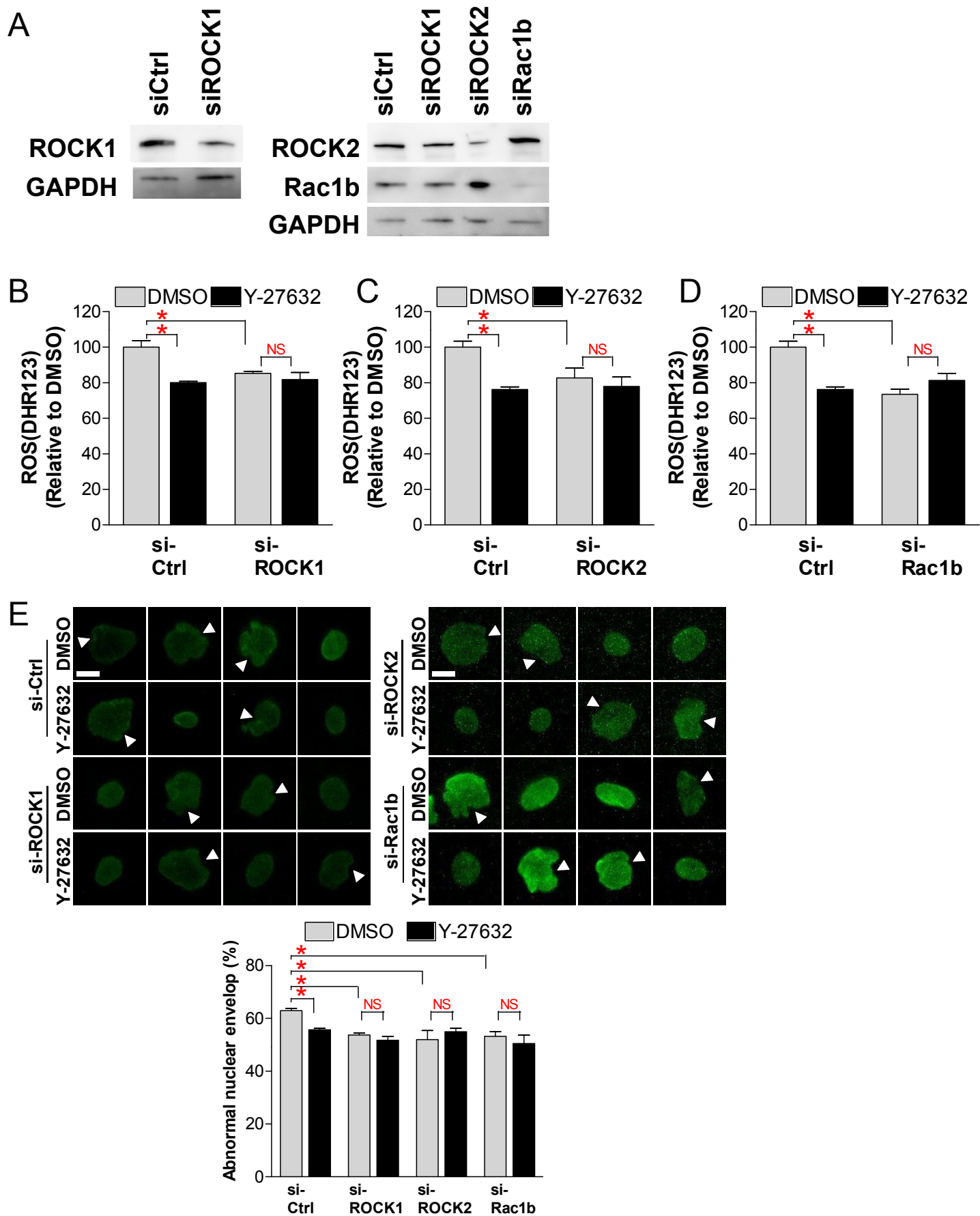

Supplement: Supplementary file 2 — Fig. S2 Effect of Y‐27632 on ROCK1, ROCK2, or Rac1b deficient HGPS fibroblasts. [file ACEL-16-541-s002.pdf]

# Supplementary Figure 3

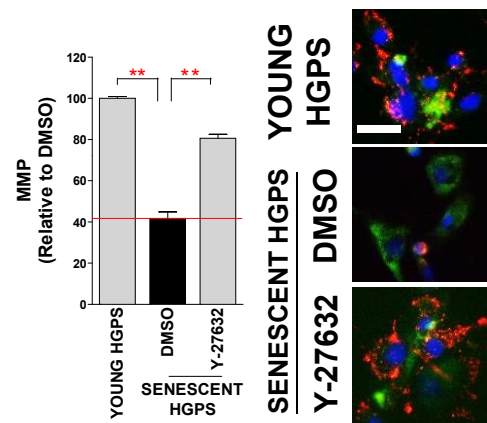

Supplement: Supplementary file 3 — Fig. S3 Effect of Y‐27632 on the recovery of MMP. [file ACEL-16-541-s003.pdf]

Supplementary Figure 4

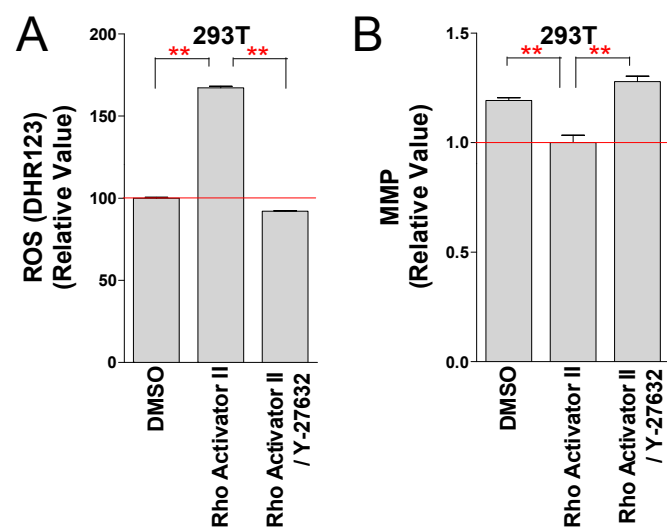

Supplement: Supplementary file 4 — Fig. S4 ROCK regulates mitochondrial function by modulating ROS levels and MMP in HEK 293T cells. [file ACEL-16-541-s004.pdf]

## Supplementary Figure 5

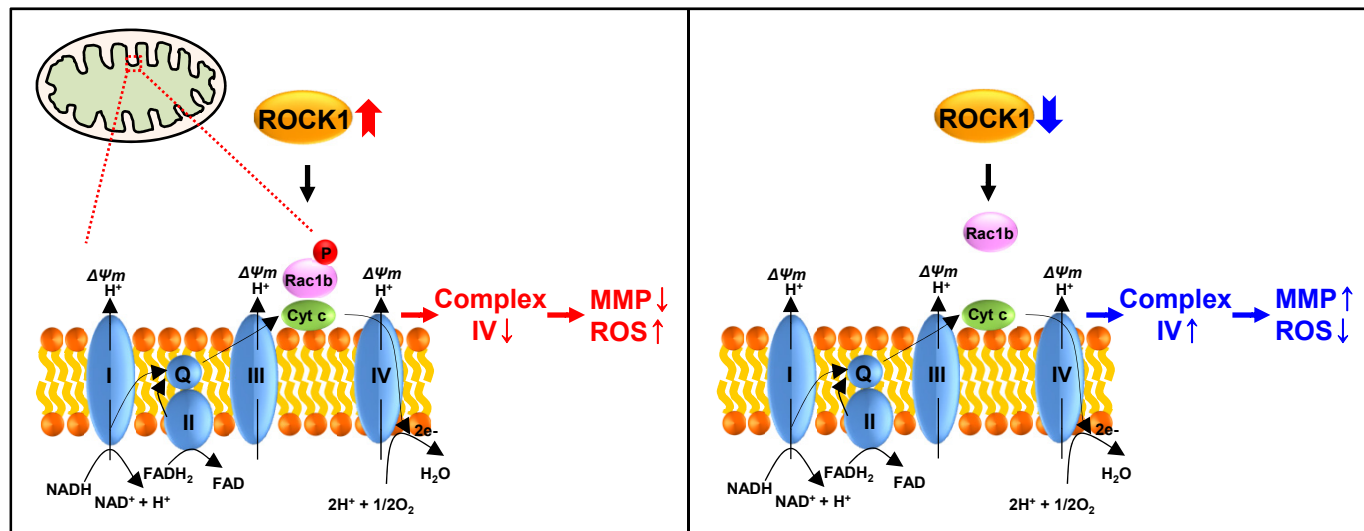

Supplement: Supplementary file 5 — Fig. S5 Proposed mechanism accounting for the regulation of mitochondrial function via the ROCK‐Rac1b‐cytochrome c axis. [file ACEL-16-541-s005.pdf]

Supplementary Figure 6

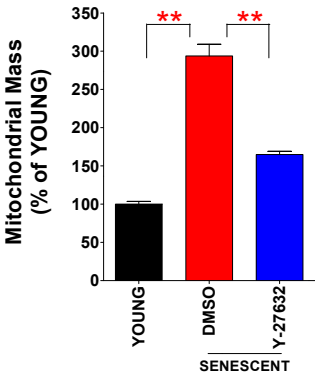

Supplement: Supplementary file 6 — Fig. S6 Effect of Y‐27632 on mitochondrial mass. [file ACEL-16-541-s006.pdf]
