# Supplementary material for: Chemical screening identifies ROCK as a target for recovering mitochondrial function in Hutchinson‐Gilford progeria syndrome
Source: Aging Cell. 2017 Mar 19;16(3):541–50. doi: 10.1111/acel.12584 (PMC5418208; doi:10.1111/acel.12584)
Supplement: Supplementary file 7 — Table S1 Detailed list of ROS levels in high‐throughput screening. [file ACEL-16-541-s007.pdf]

Supplementary Table 1. The detailed list of ROS level in high throughput screening

	Name	DNA Contents Value (Normalized by DMSO value)						Average	DHR123 VALUE (Normalized by DMSO value)						ROS Level (DHR123 Value/DNA contents)						Average	STDEV
1	*Linifanib (ABT-869)*	0.49	0.39	0.44	0.43	0.43	0.41	0.43	0.37	0.35	0.32	0.31	0.38	0.37	0.86	0.82	0.75	0.72	0.88	0.85	0.81	0.06
2	*Axitinib*	0.32	0.29	0.32	0.30	0.27	0.30	0.30	0.65	0.63	0.67	0.57	0.62	0.63	2.18	2.12	2.23	1.91	2.08	2.10	2.10	0.11
3	*Saracatinib (AZD0530)*	1.04	0.63	1.32	0.94	1.24	0.77	0.99	0.83	0.98	1.00	0.99	0.84	0.70	0.83	0.99	1.01	1.00	0.85	0.70	0.90	0.12
4	*Selumetinib (AZD6244)*	0.48	0.47	0.46	0.42	0.58	0.51	0.49	0.58	0.56	0.48	0.47	0.46	0.51	1.18	1.15	0.99	0.97	0.95	1.04	1.05	0.10
5	*BEZ235 (NVP-BEZ235, Dactolisib)*	0.51	0.54	0.52	0.47	0.55	0.29	0.48	0.47	0.61	0.55	0.55	0.58	0.62	0.98	1.27	1.15	1.14	1.21	1.30	1.17	0.11
6	*Nintedanib (BIBF 1120)*	0.24	0.22	0.24	0.23	0.28	0.22	0.24	0.52	0.51	0.43	0.49	0.48	0.49	2.18	2.13	1.81	2.04	2.03	2.04	2.04	0.13
7	*Afatinib (BIBW2992)*	0.21	0.23	0.20	0.24	0.23	0.25	0.23	0.49	0.48	0.52	0.50	0.51	0.54	2.18	2.14	2.29	2.20	2.27	2.40	2.25	0.09
8	*Bosutinib (SKI-606)*	0.45	0.44	0.52	0.49	0.34	0.39	0.44	0.70	0.66	0.65	0.73	0.70	0.93	1.59	1.51	1.49	1.68	1.61	2.12	1.66	0.23
9	*Cediranib (AZD2171)*	0.26	0.26	0.24	0.30	0.23	0.23	0.25	0.50	0.47	0.43	0.50	0.56	0.41	1.99	1.86	1.72	1.97	2.20	1.63	1.90	0.21
10	*Dovitinib (TKI-258, CHIR-258)*	0.18	0.21	0.19	0.17	0.23	0.22	0.20	0.56	0.51	0.50	0.52	0.42	0.46	2.83	2.54	2.48	2.62	2.09	2.31	2.48	0.25
11	*Canertinib (CI-1033)*	0.47	0.47	0.37	0.37	0.34	0.32	0.39	0.58	0.47	0.59	0.58	0.50	0.63	1.47	1.21	1.51	1.49	1.28	1.62	1.43	0.16
12	*PD184352 (CI-1040)*	0.28	0.30	0.29	0.28	0.27	0.31	0.29	0.44	0.44	0.35	0.40	0.39	0.42	1.51	1.52	1.21	1.39	1.34	1.44	1.40	0.11
13	*Dasatinib*	0.45	0.36	0.29	0.37	0.45	0.39	0.39	0.55	0.30	0.39	0.32	0.37	0.48	1.42	0.78	1.01	0.83	0.96	1.25	1.04	0.25
**14**	***Ridaforolimus (Deforolimus, MK-86***	0.88	1.13	0.50	0.85	0.56	0.79	0.79	0.54	0.68	0.42	0.55	0.46	0.51	0.68	0.86	0.54	0.70	0.58	0.65	0.67	0.11
**15**	***Erlotinib HCl (OSI-744)***	0.33	0.46	0.36	0.45	0.43	0.41	0.41	0.38	0.51	0.35	0.34	0.37	0.41	0.93	1.26	0.85	0.83	0.91	1.02	0.97	0.16
**16**	***Gefitinib (ZD1839)***	0.64	0.55	0.90	0.75	0.55	0.48	0.64	0.82	0.84	0.74	0.63	0.54	0.68	1.26	1.30	1.14	0.98	0.83	1.05	1.10	0.18
**17**	***Imatinib Mesylate (STI571)***	0.44	0.37	0.40	0.40	0.37	0.39	0.40	0.44	0.42	0.36	0.42	0.37	0.50	1.12	1.06	0.92	1.06	0.94	1.25	1.06	0.12
**18**	***Lapatinib (GW-572016) Ditosylate***	0.92	1.00	1.01	0.94	0.70	0.90	0.91	0.63	0.56	0.57	0.76	0.51	0.54	0.69	0.62	0.62	0.83	0.56	0.59	0.65	0.10
**19**	***Motesanib Diphosphate (AMG-706)***	0.26	0.19	0.27	0.25	0.22	0.25	0.24	0.21	0.29	0.36	0.22	0.25	0.30	0.89	1.20	1.47	0.93	1.02	1.26	1.13	0.22
**20**	***Nilotinib (AMN-107)***	0.40	0.36	0.37	0.38	0.32	0.37	0.37	0.49	0.44	0.28	0.39	0.45	0.39	1.34	1.21	0.77	1.06	1.22	1.06	1.11	0.20
**21**	***NVP-AEW541***	0.21	0.23	0.23	0.20	0.18	0.20	0.21	0.44	0.59	0.40	0.33	0.37	0.50	2.12	2.83	1.92	1.58	1.79	2.40	2.11	0.45
**22**	***Pazopanib HCl (GW786034 HCl)***	0.29	0.33	0.20	0.34	0.18	0.42	0.29	0.43	0.40	0.38	0.40	0.36	0.37	1.49	1.38	1.30	1.37	1.22	1.26	1.34	0.10
**23**	***PD0325901***	0.22	0.20	0.22	0.25	0.18	0.21	0.21	0.27	0.25	0.21	0.22	0.22	0.23	1.27	1.17	1.00	1.06	1.06	1.10	1.11	0.10
**24**	***PI-103***	0.24	0.27	0.33	0.28	0.31	0.31	0.29	0.38	0.34	0.28	0.32	0.35	0.38	1.31	1.17	0.97	1.10	1.21	1.29	1.17	0.13
**25**	***Rapamycin (Sirolimus)***	0.67	0.68	0.76	0.80	1.13	0.88	0.82	0.42	0.43	0.69	0.43	0.44	0.55	0.51	0.53	0.84	0.53	0.54	0.68	0.60	0.13
**26**	***Sorafenib Tosylate***	0.56	0.66	0.59	0.43	0.35	0.54	0.52	0.46	0.49	0.42	0.40	0.38	0.47	0.89	0.93	0.80	0.77	0.72	0.91	0.84	0.09
**27**	***Sunitinib Malate***	0.23	0.33	0.38	0.33	0.44	0.40	0.35	0.43	0.37	0.37	0.28	0.31	0.35	1.23	1.05	1.05	0.80	0.88	1.01	1.00	0.15
**28**	***Tandutinib (MLN518)***	0.44	0.55	0.37	0.56	0.43	0.42	0.46	0.41	0.42	0.39	0.45	0.34	0.36	0.89	0.90	0.85	0.97	0.74	0.79	0.86	0.08
**29**	***Temsirolimus (CCI-779, NSC 683864***	0.42	0.32	0.32	0.38	0.46	0.63	0.42	0.32	0.27	0.27	0.28	0.30	0.30	0.76	0.65	0.65	0.66	0.70	0.71	0.69	0.04
**30**	***Vandetanib (ZD6474)***	0.31	0.37	0.49	0.34	0.38	0.33	0.37	0.44	0.38	0.37	0.46	0.38	0.43	1.18	1.02	0.99	1.25	1.01	1.15	1.10	0.11
**31**	***VX-680 (Tozasertib, MK-0457)***	0.45	0.24	0.23	0.30	0.31	0.33	0.31	0.36	0.29	0.28	0.28	0.26	0.31	1.15	0.94	0.89	0.90	0.85	1.00	0.95	0.11
**32**	***Y-27632 2HCl***	1.28	1.73	1.24	1.24	1.16	1.01	1.28	0.49	0.66	0.49	0.73	0.50	0.49	0.39	0.52	0.38	0.57	0.39	0.38	0.44	0.08
**33**	***Enzastaurin (LY317615)***	0.41	0.31	0.45	0.43	0.36	0.40	0.40	0.30	0.26	0.26	0.27	0.27	0.38	0.77	0.65	0.65	0.69	0.70	0.95	0.74	0.11
**34**	***AC480 (BMS-599626)***	1.00	1.12	0.94	0.91	1.00	0.92	0.98	0.46	0.46	0.40	0.42	0.43	0.43	0.47	0.47	0.40	0.43	0.44	0.44	0.44	0.02
**35**	***Masitinib (AB1010)***	0.39	0.42	0.40	0.34	0.37	0.32	0.37	0.55	0.53	0.47	0.49	0.50	0.44	1.49	1.42	1.27	1.31	1.34	1.17	1.33	0.11
**36**	***GDC-0941***	0.40	0.49	0.44	0.42	0.33	0.48	0.43	0.37	0.32	0.35	0.39	0.35	0.38	0.86	0.75	0.81	0.91	0.83	0.90	0.84	0.06
**37**	***SL-327***	0.62	0.71	0.72	0.79	0.50	0.56	0.65	0.47	0.50	0.55	0.49	0.48	0.61	0.72	0.76	0.84	0.75	0.74	0.93	0.79	0.08
**38**	***Crizotinib (PF-02341066)***	0.38	0.48	0.47	0.47	0.44	0.47	0.45	0.35	0.36	0.39	0.32	0.37	0.38	0.78	0.80	0.86	0.71	0.81	0.85	0.80	0.05
**39**	***PHA-665752***	0.22	0.62	0.69	0.70	0.67	0.25	0.53	0.25	0.39	1.41	0.33	0.31	0.47	0.48	0.75	2.68	0.62	0.59	0.90	1.00	0.84
**40**	***ZSTK474***	0.91	0.68	0.37	0.54	0.59	0.46	0.59	0.31	0.39	0.32	0.35	0.32	0.30	0.53	0.65	0.54	0.59	0.54	0.51	0.56	0.05
**41**	***SB216763***	1.05	1.22	0.80	0.97	1.06	1.01	1.02	0.65	0.76	0.80	0.82	0.85	0.75	0.64	0.75	0.79	0.81	0.84	0.74	0.76	0.07
**42**	***SB203580***	1.46	2.24	1.88	1.03	1.71	1.93	1.71	0.89	0.90	0.79	1.03	0.84	0.80	0.52	0.53	0.46	0.61	0.49	0.47	0.51	0.05
**43**	***SB202190 (FHPI)***	2.19	1.06	2.22	2.85	2.34	1.59	2.04	1.38	1.12	1.30	1.26	1.16	1.37	0.68	0.55	0.63	0.62	0.57	0.67	0.62	0.05

44	*MK-2206 2HCl*	1.06	0.66	0.67	0.99	0.75	0.82	0.83	0.50	0.79	0.49	0.61	0.55	0.66	0.61	0.95	0.59	0.74	0.67	0.80	0.72	0.14
45	*SU11274*	0.84	0.82	1.27	0.49	0.62	0.75	0.80	0.57	0.52	0.57	0.51	0.47	0.49	0.71	0.65	0.71	0.64	0.59	0.62	0.65	0.05
46	*Brivanib (BMS-540215)*	1.68	1.08	1.45	1.77	0.92	1.16	1.34	0.68	0.90	0.78	1.07	0.92	0.75	0.51	0.67	0.58	0.80	0.69	0.56	0.63	0.10
47	*NVP-ADW742*	0.15	0.18	0.20	0.19	0.19	0.18	0.18	0.35	0.26	0.28	0.25	0.27	0.30	1.90	1.42	1.52	1.40	1.50	1.62	1.56	0.19
48	*OSI-906 (Linsitinib)*	1.17	1.16	1.37	1.27	1.00	1.50	1.25	0.92	0.89	0.85	0.96	0.81	0.83	0.74	0.71	0.68	0.77	0.65	0.67	0.71	0.05
49	*KU-55933 (ATM Kinase Inhibitor)*	1.15	1.85	0.92	1.38	1.46	0.71	1.24	1.19	1.87	1.78	1.58	1.46	1.44	0.96	1.50	1.43	1.27	1.17	1.16	1.25	0.20
50	*GSK1904529A*	0.77	0.77	0.73	1.00	0.93	0.91	0.85	0.61	0.62	0.51	0.67	0.46	0.57	0.72	0.72	0.60	0.79	0.54	0.67	0.67	0.09
51	*PF-04217903*	1.47	1.40	1.14	1.24	0.78	1.05	1.18	1.41	1.30	1.04	1.31	1.50	1.12	1.20	1.10	0.88	1.11	1.27	0.95	1.08	0.15
52	*MLN8054*	0.86	0.73	0.86	0.98	0.62	0.72	0.80	0.92	0.79	0.73	0.92	0.76	0.74	1.15	1.00	0.92	1.15	0.96	0.93	1.02	0.11
53	*Vatalanib (PTK787) 2HCl*	0.46	0.51	0.36	0.43	0.31	0.32	0.40	0.34	0.58	0.64	0.52	0.35	0.35	0.85	1.46	1.60	1.31	0.87	0.89	1.16	0.34
54	*U0126-EtOH*	0.93	1.08	0.73	0.85	0.68	0.61	0.81	0.34	0.29	0.32	0.29	0.27	0.31	0.42	0.36	0.39	0.35	0.33	0.38	0.37	0.03
55	*ZM 447439*	0.46	0.42	0.57	0.39	0.66	0.46	0.50	0.82	0.73	0.68	0.85	0.71	0.52	1.66	1.47	1.37	1.72	1.43	1.05	1.45	0.24
56	*GDC-0879*	2.67	1.17	2.02	1.82	1.76	1.87	1.88	1.13	1.55	1.64	1.55	1.66	1.64	0.60	0.82	0.87	0.82	0.88	0.87	0.81	0.11
57	*LY294002*	0.96	0.76	0.93	1.07	0.83	0.83	0.90	0.68	0.85	1.10	1.19	0.87	0.55	0.75	0.94	1.23	1.33	0.97	0.62	0.97	0.27
58	*OSU-03012 (AR-12)*	0.74	0.75	0.98	1.10	0.92	0.39	0.82	0.30	0.52	0.46	0.43	0.39	0.56	0.37	0.64	0.56	0.53	0.48	0.68	0.54	0.11
59	*Danusertib (PHA-739358)*	0.20	0.20	0.15	0.23	0.17	0.18	0.19	0.29	0.25	0.24	0.24	0.22	0.30	1.56	1.31	1.29	1.29	1.15	1.60	1.37	0.18
60	*TAE684 (NVP-TAE684)*	0.17	0.13	0.15	0.17	0.09	0.16	0.15	0.17	0.17	0.23	0.19	0.19	0.24	1.14	1.15	1.60	1.30	1.28	1.66	1.36	0.22
61	*BI 2536*	0.26	0.24	0.21	0.20	0.26	0.28	0.24	0.33	0.35	0.42	0.37	0.42	0.42	1.36	1.43	1.73	1.51	1.72	1.72	1.58	0.17
62	*Foretinib (GSK1363089)*	0.22	0.27	0.23	0.28	0.22	0.24	0.24	0.36	0.37	0.28	0.37	0.33	0.46	1.49	1.50	1.14	1.52	1.37	1.87	1.48	0.24
63	*SGX-523*	0.75	0.72	0.91	0.83	0.90	0.69	0.80	0.52	0.52	0.58	0.61	0.66	0.68	0.65	0.64	0.72	0.76	0.82	0.86	0.74	0.09
64	*GSK690693*	0.44	0.52	0.51	0.68	0.57	0.58	0.55	0.45	0.59	0.55	0.46	0.52	0.63	0.82	1.07	1.00	0.83	0.95	1.15	0.97	0.13
65	*JNJ-38877605*	0.57	0.80	0.73	0.71	0.98	0.66	0.74	0.81	0.68	0.64	0.67	0.65	0.66	1.09	0.91	0.86	0.91	0.87	0.89	0.92	0.08
66	*Palbociclib (PD-0332991) HCl*	0.67	0.39	0.50	0.58	0.47	0.59	0.53	0.57	0.51	0.54	0.44	0.48	0.58	1.07	0.96	1.01	0.83	0.90	1.09	0.98	0.10
67	*Triciribine*	0.60	0.54	0.54	0.83	0.60	0.60	0.62	0.38	0.40	0.47	0.39	0.44	0.38	0.62	0.64	0.77	0.63	0.72	0.62	0.67	0.06
68	*XL147*	0.84	0.88	0.97	0.96	0.79	0.73	0.86	0.58	0.61	0.54	0.59	0.50	0.60	0.67	0.71	0.63	0.68	0.58	0.69	0.66	0.05
69	*Cabozantinib (XL184, BMS-907351)*	0.33	0.26	0.36	0.25	0.33	0.30	0.30	0.39	0.44	0.56	0.34	0.41	0.41	1.29	1.45	1.85	1.13	1.35	1.34	1.40	0.24
70	*Everolimus (RAD001)*	1.07	1.04	0.92	0.70	0.71	0.91	0.89	0.72	0.62	0.63	0.63	0.51	0.67	0.81	0.69	0.71	0.71	0.57	0.75	0.71	0.08
71	*BMS-754807*	0.42	0.36	0.34	0.27	0.37	0.39	0.36	0.46	0.41	0.47	0.37	0.37	0.45	1.28	1.13	1.30	1.02	1.04	1.26	1.17	0.12
72	*Alisertib (MLN8237)*	0.79	0.77	0.84	0.68	0.62	0.78	0.75	0.64	0.76	0.73	0.78	0.72	0.72	0.85	1.03	0.99	1.04	0.97	0.97	0.98	0.07
73	*AT9283*	0.22	0.19	0.21	0.23	0.20	0.27	0.22	0.24	0.21	0.29	0.19	0.27	0.26	1.08	0.97	1.33	0.87	1.24	1.17	1.11	0.17
74	*Brivanib Alaninate (BMS-582664)*	0.92	1.44	1.51	1.15	1.25	0.98	1.21	0.94	1.04	0.87	1.07	0.87	0.90	0.78	0.86	0.72	0.89	0.72	0.74	0.79	0.07
75	*AG-490 (Tyrphostin B42)*	0.92	0.48	0.77	0.74	0.72	0.62	0.71	0.50	0.34	0.37	0.31	0.26	0.29	0.70	0.48	0.52	0.43	0.37	0.42	0.49	0.12
76	*SNS-032 (BMS-387032)*	0.29	0.24	0.28	0.21	0.18	0.28	0.25	0.31	0.31	0.26	0.31	0.29	0.28	1.24	1.25	1.05	1.26	1.18	1.13	1.18	0.08
77	*Barasertib (AZD1152-HQPA)*	0.53	0.52	0.61	0.52	0.51	0.53	0.53	0.37	0.42	0.37	0.37	0.42	0.39	0.70	0.79	0.69	0.69	0.78	0.72	0.73	0.05
78	*PLX-4720*	0.85	0.92	0.70	0.46	0.56	0.50	0.66	0.93	0.38	0.49	0.33	0.30	0.23	1.40	0.58	0.74	0.50	0.45	0.35	0.67	0.38
79	*Roscovitine (Seliciclib,CYC202)*	0.25	0.24	0.26	0.26	0.22	0.20	0.24	0.26	0.28	0.27	0.21	0.28	0.26	1.10	1.17	1.12	0.90	1.19	1.07	1.09	0.11
80	*SNS-314 Mesylate*	0.34	0.39	0.50	0.42	0.43	0.35	0.41	0.37	0.39	0.43	0.47	0.40	0.45	0.92	0.96	1.06	1.15	0.98	1.11	1.03	0.09
81	*Lenvatinib (E7080)*	0.29	0.25	0.28	0.27	0.26	0.23	0.26	0.30	0.25	0.26	0.26	0.29	0.29	1.16	0.94	0.98	0.99	1.12	1.11	1.05	0.09
82	*CP-724714*	0.76	0.63	0.82	0.66	0.58	0.58	0.67	0.59	0.63	0.70	0.67	0.65	0.69	0.87	0.94	1.03	1.00	0.97	1.02	0.97	0.06
83	*TGX-221*	0.55	0.40	0.77	0.39	0.42	0.30	0.47	0.41	0.41	0.50	0.40	0.36	0.33	0.86	0.87	1.07	0.84	0.76	0.71	0.85	0.12
84	*WZ3146*	0.22	0.19	0.28	0.33	0.21	0.21	0.24	0.26	0.20	0.18	0.22	0.23	0.27	1.08	0.83	0.76	0.91	0.97	1.11	0.94	0.14
85	*CYC116*	0.67	0.64	0.50	0.64	0.53	0.55	0.59	0.39	0.40	0.41	0.37	0.37	0.47	0.66	0.68	0.70	0.63	0.63	0.80	0.68	0.06
86	*WZ4002*	0.59	0.83	0.47	0.74	0.58	0.27	0.58	0.51	0.55	0.35	0.40	0.34	0.31	0.87	0.94	0.60	0.68	0.58	0.54	0.70	0.17
87	*PD98059*	1.08	0.93	0.93	0.92	1.23	1.08	1.03	1.01	0.95	1.03	1.04	1.02	1.11	0.98	0.92	1.00	1.01	0.99	1.08	1.00	0.05
88	*Regorafenib (BAY 73-4506)*	0.48	0.73	0.58	0.47	0.42	0.57	0.54	0.57	0.48	0.28	0.45	0.30	0.34	1.05	0.88	0.52	0.83	0.56	0.63	0.75	0.21
89	*WZ8040*	0.99	0.43	0.21	0.37	0.22	0.22	0.41	0.42	0.31	0.29	0.35	0.39	0.34	1.04	0.77	0.70	0.85	0.95	0.84	0.86	0.12

90	*ENMD-2076*	0.26	0.28	0.31	0.25	0.24	0.28	0.27	0.62	0.46	0.39	0.53	0.47	0.40	2.27	1.68	1.45	1.94	1.75	1.48	1.76	0.31
91	*CUDC-101*	0.57	0.38	0.24	0.26	0.31	0.30	0.34	0.56	0.39	0.36	0.29	0.43	0.31	1.62	1.13	1.06	0.83	1.25	0.91	1.13	0.28
92	*PIK-75*	0.28	0.21	0.24	0.23	0.24	0.24	0.24	0.59	0.47	0.41	0.50	0.36	0.45	2.46	1.96	1.69	2.09	1.51	1.87	1.93	0.33
93	*Tivozanib (AV-951)*	0.51	0.36	0.35	0.38	0.33	0.26	0.37	0.41	0.46	0.47	0.38	0.40	0.46	1.12	1.26	1.27	1.05	1.10	1.25	1.18	0.10
94	*YM201636*	0.58	0.55	0.71	0.57	0.40	0.39	0.53	0.45	0.38	0.51	0.49	0.37	0.41	0.85	0.72	0.97	0.92	0.70	0.77	0.82	0.11
95	*OSI-930*	0.53	0.42	0.43	0.35	0.35	0.47	0.42	0.52	0.45	0.51	0.39	0.39	0.29	1.23	1.05	1.21	0.92	0.93	0.67	1.00	0.21
96	*KU-0063794*	0.36	0.56	0.55	0.47	0.52	0.42	0.48	0.36	0.32	0.43	0.43	0.37	0.50	0.75	0.66	0.90	0.89	0.78	1.04	0.84	0.13
97	*AG-1024*	1.04	1.33	1.13	1.27	1.32	1.02	1.19	1.11	1.05	1.40	1.39	1.45	1.29	0.94	0.88	1.18	1.17	1.22	1.09	1.08	0.14
98	*Amuvatinib (MP-470)*	0.74	0.72	0.67	0.46	0.66	0.35	0.60	0.60	1.13	0.49	0.39	0.45	0.60	1.01	1.88	0.82	0.65	0.74	1.00	1.02	0.45
99	*JNJ-7706621*	0.86	1.30	0.82	1.11	0.85	0.89	0.97	1.07	1.16	1.16	1.14	1.11	1.11	1.10	1.19	1.20	1.17	1.14	1.14	1.16	0.04
100	*PD173074*	0.40	0.51	0.34	0.21	0.37	0.25	0.35	0.47	0.39	0.31	0.30	0.31	0.33	1.37	1.12	0.90	0.86	0.89	0.94	1.02	0.20
101	*WYE-354*	0.43	0.49	0.64	0.55	0.50	0.47	0.51	0.68	0.40	0.55	0.36	0.57	0.36	1.32	0.78	1.07	0.71	1.11	0.70	0.95	0.25
102	*Vemurafenib (PLX4032, RG7204)*	0.90	1.11	0.83	0.89	1.18	0.96	0.98	1.01	1.14	0.84	1.02	0.88	0.83	1.03	1.17	0.86	1.05	0.90	0.85	0.98	0.13
103	*BX-795*	0.36	0.29	0.31	0.28	0.21	0.34	0.30	0.36	0.29	0.26	0.34	0.33	0.31	1.21	0.96	0.86	1.14	1.12	1.03	1.05	0.13
104	*BX-912*	0.27	0.26	0.27	0.30	0.22	0.21	0.25	0.31	0.29	0.20	0.31	0.21	0.28	1.21	1.15	0.80	1.23	0.82	1.12	1.05	0.19
105	*Zoledronic Acid*	1.04	1.25	0.94	1.22	1.18	1.10	1.12	0.84	0.93	0.62	0.87	0.71	0.58	0.74	0.83	0.55	0.78	0.64	0.52	0.68	0.13
106	*Genistein*	1.04	0.96	0.87	0.59	0.73	0.86	0.84	0.65	0.67	0.58	0.68	0.60	0.59	0.77	0.79	0.69	0.80	0.72	0.70	0.74	0.05
107	*TG100-115*	1.43	1.13	0.93	1.42	0.84	1.12	1.15	0.85	0.86	0.76	0.77	0.60	0.65	0.74	0.75	0.66	0.68	0.53	0.57	0.65	0.09
108	*GSK1059615*	0.40	0.32	0.34	0.30	0.20	0.21	0.30	0.34	0.26	0.28	0.33	0.24	0.35	1.14	0.88	0.94	1.10	0.82	1.20	1.01	0.15
109	*MGCD-265*	0.62	0.50	0.65	0.56	0.54	0.64	0.58	0.54	0.44	0.49	0.41	0.48	0.45	0.93	0.75	0.84	0.71	0.82	0.76	0.80	0.08
110	*Rigosertib (ON-01910)*	0.51	0.62	0.52	0.62	0.67	0.61	0.59	0.40	0.45	0.44	0.50	0.44	0.74	0.68	0.76	0.74	0.83	0.73	1.25	0.83	0.21
111	*Ki8751*	0.83	0.91	0.72	0.72	0.78	0.89	0.81	0.57	0.68	0.63	0.82	0.85	0.81	0.71	0.83	0.77	1.02	1.05	0.99	0.89	0.14
112	*Ruxolitinib (INCB018424)*	0.80	0.85	0.66	0.66	0.63	0.68	0.71	0.57	0.55	0.52	0.56	0.56	0.55	0.79	0.78	0.72	0.79	0.79	0.78	0.77	0.03
113	*Pelitinib (EKB-569)*	0.27	0.27	0.26	0.26	0.25	0.36	0.28	0.75	0.36	0.47	0.56	0.77	0.48	2.70	1.31	1.69	2.01	2.78	1.74	2.04	0.59
114	*Aurora A Inhibitor I*	0.78	0.71	0.70	0.55	0.58	0.47	0.63	0.60	0.48	0.46	0.84	0.62	0.67	0.95	0.75	0.73	1.32	0.98	1.06	0.97	0.22
115	*PHA-680632*	0.37	0.34	0.35	0.30	0.33	0.39	0.35	0.48	0.44	0.44	0.39	0.43	0.41	1.38	1.28	1.27	1.11	1.24	1.19	1.25	0.09
116	*VX-745*	1.59	1.77	1.93	1.56	1.87	1.47	1.70	1.34	1.22	1.15	1.14	1.43	0.91	0.79	0.72	0.68	0.67	0.84	0.54	0.71	0.11
117	*Thiazovivin*	0.64	0.64	0.51	0.55	0.51	0.49	0.56	0.53	0.59	0.58	0.51	0.51	0.53	0.95	1.05	1.05	0.92	0.92	0.95	0.97	0.06
118	*SP600125*	0.65	0.78	0.73	0.50	0.56	0.47	0.61	0.56	0.58	0.45	0.53	0.54	0.47	0.91	0.95	0.73	0.86	0.88	0.76	0.85	0.08
119	*AZD6482*	0.56	0.64	0.62	0.53	0.50	0.59	0.57	0.47	0.48	0.42	0.43	0.53	0.50	0.82	0.84	0.73	0.75	0.92	0.88	0.82	0.07
120	*TSU-68 (SU6668, Orantinib)*	1.32	1.31	1.30	0.90	0.76	0.71	1.05	0.58	0.57	0.51	0.58	0.50	0.67	0.55	0.54	0.49	0.55	0.48	0.64	0.54	0.06
121	*GSK429286A*	1.28	1.86	1.55	1.11	1.51	1.27	1.43	2.06	1.69	1.59	1.44	1.41	1.35	1.44	1.18	1.11	1.00	0.99	0.94	1.11	0.18
122	*Pimasertib (AS-703026)*	0.36	0.35	0.32	0.36	0.38	0.28	0.34	0.45	0.48	0.39	0.35	0.34	0.41	1.33	1.40	1.13	1.01	0.99	1.19	1.17	0.17
123	*HMN-214*	0.46	0.52	0.59	0.53	0.55	0.57	0.54	0.74	0.58	0.58	0.62	0.53	0.52	1.38	1.07	1.08	1.15	0.99	0.96	1.11	0.15
124	*AEE788 (NVP-AEE788)*	0.40	0.42	0.32	0.27	0.42	0.30	0.35	0.46	0.45	0.41	0.70	0.37	0.50	1.29	1.26	1.16	1.96	1.03	1.40	1.35	0.32
125	*PHA-793887*	0.25	0.24	0.29	0.24	0.21	0.25	0.25	0.39	0.44	0.42	0.44	0.44	0.44	1.55	1.78	1.69	1.76	1.79	1.76	1.72	0.09
126	*PIK-93*	0.43	0.48	0.46	0.42	0.52	0.48	0.46	0.47	0.46	0.60	0.40	0.41	0.51	1.02	0.99	1.30	0.87	0.88	1.10	1.03	0.16
127	*Ponatinib (AP24534)*	0.26	0.23	0.27	0.29	0.23	0.24	0.26	0.72	0.48	0.62	0.63	0.61	0.75	2.81	1.87	2.42	2.49	2.40	2.93	2.49	0.37
128	*LY2228820*	1.32	1.64	0.93	1.21	0.81	1.19	1.18	0.79	0.80	0.65	0.76	0.74	1.01	0.66	0.67	0.55	0.65	0.63	0.85	0.67	0.10
129	*CCT129202*	0.26	0.24	0.28	0.20	0.23	0.24	0.24	1.06	1.11	0.76	0.67	0.71	0.97	4.37	4.57	3.16	2.78	2.95	4.01	3.64	0.77
130	*SAR245409 (XL765)*	1.57	1.56	1.26	1.87	1.41	1.30	1.50	0.96	1.00	0.92	0.85	0.96	0.90	0.64	0.67	0.62	0.57	0.64	0.60	0.62	0.04
131	*AT7519*	0.28	0.22	0.28	0.26	0.21	0.22	0.25	0.64	0.40	0.46	0.45	0.31	0.45	2.62	1.65	1.89	1.85	1.26	1.83	1.85	0.44
132	*Quizartinib (AC220)*	0.37	0.32	0.35	0.35	0.36	0.33	0.35	0.41	0.44	0.46	0.41	0.54	0.67	1.19	1.27	1.33	1.19	1.55	1.91	1.41	0.28
133	*Hesperadin*	0.44	0.45	0.39	0.36	0.31	0.35	0.38	0.48	0.43	0.50	0.38	0.54	0.38	1.24	1.12	1.30	1.00	1.42	1.00	1.18	0.17
134	*BIX 02188*	0.59	0.56	0.55	0.68	0.58	0.70	0.61	0.53	0.46	0.43	0.54	0.42	0.57	0.87	0.75	0.71	0.88	0.69	0.94	0.81	0.10
135	*BIX 02189*	0.46	0.39	0.59	0.47	0.38	0.57	0.48	0.50	0.44	0.55	0.47	0.43	0.49	1.05	0.91	1.16	0.98	0.90	1.03	1.00	0.10

136	AZD7762	0.28	0.33	0.30	0.29	0.24	0.30	0.29	0.44	0.50	0.48	0.41	0.35	0.37	1.54	1.75	1.66	1.44	1.22	1.30	1.48	0.20
137	R406 (free base)	0.28	0.34	0.38	0.27	0.36	0.38	0.33	0.38	0.32	0.37	0.42	0.39	0.45	1.15	0.95	1.11	1.25	1.16	1.34	1.16	0.13
138	CP-673451	0.26	0.29	0.26	0.34	0.28	0.26	0.28	0.38	0.43	0.44	0.44	0.40	0.44	1.34	1.51	1.55	1.55	1.41	1.54	1.48	0.09
139	AZD8055	0.41	0.38	0.57	0.42	0.35	0.44	0.43	0.42	0.34	0.41	0.41	0.39	0.34	0.99	0.80	0.96	0.96	0.92	0.79	0.90	0.09
140	PHT-427	0.44	0.63	0.82	0.66	0.57	0.79	0.65	0.39	0.62	0.56	0.51	0.45	0.68	0.59	0.95	0.86	0.78	0.69	1.04	0.82	0.17
141	KRN 633	0.44	0.52	0.39	0.44	0.47	0.40	0.44	0.51	0.37	0.35	0.40	0.46	0.39	1.16	0.84	0.78	0.89	1.04	0.89	0.93	0.14
142	AT7867	0.43	0.43	0.37	0.33	0.37	0.41	0.39	0.48	0.52	0.36	0.44	0.44	0.44	1.22	1.32	0.93	1.13	1.12	1.13	1.14	0.13
143	BMS-777607	0.66	0.67	0.58	0.46	0.44	0.38	0.53	0.61	0.53	0.53	0.55	0.57	0.55	1.14	1.00	1.01	1.03	1.08	1.04	1.05	0.05
144	PD318088	0.43	0.45	0.48	0.40	0.39	0.56	0.45	0.43	0.64	0.39	0.40	0.39	0.58	0.95	1.42	0.86	0.89	0.85	1.28	1.04	0.24
145	KU-60019	1.22	1.34	1.37	1.51	0.87	0.83	1.19	0.98	1.18	1.42	1.38	1.35	1.25	0.83	0.99	1.19	1.16	1.13	1.05	1.06	0.14
146	BS-181 HCl	1.13	1.08	1.20	1.32	1.11	1.07	1.15	1.32	1.24	0.92	0.96	0.91	0.89	1.15	1.08	0.80	0.83	0.79	0.77	0.90	0.17
147	Fasudil (HA-1077) HCl	1.21	1.35	1.55	1.08	1.11	1.26	1.26	1.48	2.27	2.29	2.26	1.72	1.98	1.18	1.80	1.82	1.79	1.37	1.57	1.59	0.27
148	BIRB 796 (Doramapimod)	2.74	1.50	1.71	1.65	2.46	2.12	2.03	1.12	1.13	1.23	1.17	1.07	1.15	0.55	0.55	0.61	0.57	0.53	0.57	0.56	0.03
149	Tie2 kinase inhibitor	1.27	1.69	1.60	1.79	1.25	1.43	1.50	1.07	0.88	0.85	1.05	1.36	0.86	0.71	0.59	0.57	0.70	0.90	0.57	0.67	0.13
150	H 89 2HCl	1.17	1.50	1.14	1.20	1.68	2.50	1.53	2.02	1.52	1.58	1.42	1.58	1.85	1.32	1.00	1.03	0.93	1.03	1.21	1.08	0.15
151	TWS119	0.32	0.32	0.28	0.33	0.28	0.29	0.30	0.36	0.40	0.40	0.37	0.40	0.43	1.20	1.32	1.32	1.22	1.32	1.42	1.30	0.08
152	Acadesine	1.18	1.01	1.54	1.22	1.83	1.58	1.40	0.99	1.00	0.91	0.92	1.17	1.41	0.71	0.72	0.65	0.66	0.84	1.01	0.77	0.14
153	PF-573228	0.51	0.48	0.50	0.46	0.43	0.52	0.48	0.57	0.58	0.53	0.56	0.56	0.49	1.18	1.19	1.10	1.17	1.17	1.01	1.14	0.07
154	BMS-265246	0.54	0.41	0.37	0.37	0.34	0.46	0.42	0.39	0.37	0.39	0.40	0.36	0.44	0.94	0.88	0.93	0.97	0.85	1.05	0.94	0.07
155	AZD8330	0.30	0.26	0.24	0.21	0.24	0.24	0.25	0.32	0.31	0.31	0.30	0.28	0.31	1.28	1.25	1.24	1.19	1.12	1.27	1.22	0.06
156	Neratinib (HKI-272)	0.27	0.26	0.27	0.25	0.23	0.26	0.26	0.42	0.34	0.35	0.40	0.35	0.63	1.64	1.32	1.36	1.53	1.33	2.43	1.60	0.42
157	KW-2449	0.28	0.22	0.29	0.29	0.34	0.35	0.29	0.23	0.31	0.28	0.35	0.46	0.42	0.78	1.04	0.97	1.20	1.57	1.42	1.16	0.29
158	RAF265 (CHIR-265)	0.75	0.83	0.79	0.70	0.74	0.70	0.75	0.24	0.25	0.27	0.37	0.23	0.31	0.31	0.34	0.35	0.49	0.30	0.41	0.37	0.07
159	PF-4708671	0.84	0.87	1.00	0.74	1.00	0.77	0.87	0.59	0.52	0.55	0.52	0.57	0.71	0.67	0.60	0.63	0.60	0.66	0.81	0.66	0.08
160	LY2784544	0.39	0.31	0.35	0.28	0.29	0.31	0.32	0.32	0.32	0.32	0.22	0.29	0.23	0.99	0.99	1.00	0.69	0.89	0.73	0.88	0.14
161	BGJ398 (NVP-BGJ398)	0.29	0.27	0.31	0.27	0.28	0.38	0.30	0.23	0.27	0.20	0.28	0.29	0.35	0.78	0.91	0.66	0.92	0.95	1.16	0.90	0.17
162	AST-1306	0.60	1.02	0.69	1.05	0.67	1.00	0.84	0.56	0.56	0.62	0.64	0.66	0.41	0.67	0.67	0.74	0.77	0.78	0.49	0.69	0.11
163	AZD8931 (Sapitinib)	0.73	0.76	0.73	0.72	0.67	0.67	0.71	0.60	0.43	0.53	0.41	0.50	0.68	0.84	0.61	0.74	0.58	0.70	0.95	0.74	0.14
164	GSK461364	0.49	0.45	0.46	0.45	0.37	0.49	0.45	0.43	0.33	0.26	0.41	0.37	0.38	0.96	0.75	0.58	0.91	0.83	0.85	0.81	0.14
165	R406	0.30	0.25	0.32	0.26	0.33	0.24	0.28	0.20	0.18	0.23	0.27	0.23	0.27	0.70	0.64	0.80	0.96	0.81	0.97	0.81	0.13
166	SGI-1776 free base	0.23	0.20	0.22	0.23	0.21	0.21	0.22	0.30	0.34	0.21	0.29	0.20	0.27	1.39	1.58	0.97	1.34	0.93	1.23	1.24	0.25
167	BMS-794833	0.38	0.41	0.42	0.36	0.31	0.44	0.39	0.40	0.31	0.30	0.36	0.29	0.31	1.03	0.79	0.78	0.93	0.74	0.81	0.84	0.11
168	NVP-BHG712	0.34	0.31	0.28	0.26	0.26	0.27	0.29	0.19	0.19	0.19	0.21	0.22	0.29	0.66	0.66	0.66	0.74	0.75	1.00	0.75	0.13
169	OSI-420	0.51	0.47	0.60	0.45	0.46	0.58	0.51	0.53	0.48	0.45	0.49	0.51	0.55	1.04	0.93	0.87	0.96	0.99	1.07	0.98	0.07
170	PIK-293	1.02	1.18	0.85	1.17	1.43	1.28	1.16	1.14	1.34	1.07	1.11	1.24	1.15	0.98	1.16	0.92	0.96	1.07	0.99	1.02	0.09
171	AZ 960	0.47	0.50	0.48	0.49	0.56	0.53	0.50	0.51	0.49	0.48	0.48	0.44	0.48	1.01	0.98	0.96	0.95	0.88	0.95	0.96	0.04
172	Mubritinib (TAK 165)	0.60	0.80	0.76	0.71	0.79	0.78	0.74	0.53	0.51	0.38	0.50	0.46	0.48	0.72	0.68	0.51	0.67	0.63	0.65	0.64	0.07
173	PP242	0.83	0.80	0.53	0.82	0.72	0.83	0.76	0.52	0.43	0.46	0.49	0.48	0.61	0.69	0.57	0.61	0.64	0.64	0.81	0.66	0.08
174	CYT387	0.40	0.56	0.47	0.55	0.48	0.47	0.49	0.29	0.34	0.30	0.26	0.33	0.31	0.59	0.70	0.61	0.54	0.67	0.63	0.62	0.06
175	SB590885	0.27	0.29	0.30	0.24	0.28	0.31	0.28	0.35	0.34	0.35	0.33	0.39	0.33	1.23	1.20	1.23	1.19	1.40	1.18	1.24	0.08
176	Apatinib	0.50	0.53	0.44	0.53	0.44	0.46	0.48	0.54	0.52	0.46	0.48	0.53	0.48	1.11	1.09	0.95	1.00	1.09	0.99	1.04	0.07
177	CAL-101 (Idelalisib, GS-1101)	1.05	1.46	1.61	1.60	1.33	1.27	1.39	1.26	1.54	1.66	1.48	1.68	1.48	0.91	1.11	1.20	1.06	1.21	1.06	1.09	0.11
178	PIK-294	0.66	0.60	0.75	0.85	0.96	0.65	0.75	0.79	0.73	0.80	0.72	0.76	0.90	1.06	0.98	1.07	0.97	1.01	1.21	1.05	0.09
179	Telatinib	0.46	0.45	0.41	0.44	0.33	0.31	0.40	0.69	0.65	0.59	0.59	0.61	0.61	1.73	1.63	1.49	1.49	1.53	1.52	1.57	0.09
180	Volasertib (BI 6727)	0.26	0.26	0.23	0.25	0.24	0.26	0.25	0.36	0.30	0.42	0.34	0.31	0.34	1.44	1.20	1.67	1.35	1.23	1.36	1.38	0.17
181	Palomid 529 (P529)	1.05	1.23	0.96	0.93	0.97	0.81	0.99	0.70	0.65	0.70	0.79	0.83	0.75	0.71	0.66	0.71	0.79	0.84	0.75	0.74	0.07

182	Degrasyn (WP1130)	1.01	1.23	1.23	1.35	1.37	1.23	1.24	1.47	1.00	1.03	1.13	0.84	0.97	1.19	0.81	0.83	0.91	0.68	0.79	0.87	0.17
183	BKM120 (NVP-BKM120, Buparlisib)	0.45	0.40	0.50	0.45	0.56	0.47	0.47	0.56	0.53	0.47	0.47	0.61	0.55	1.17	1.11	0.99	0.99	1.29	1.17	1.12	0.12
184	Asiatic Acid	1.12	1.09	0.89	0.79	0.88	0.66	0.90	1.13	1.01	1.93	1.23	1.04	1.45	1.25	1.12	2.14	1.36	1.15	1.60	1.43	0.39
185	Honokiol	1.32	1.09	1.06	1.28	1.13	1.37	1.21	0.82	0.88	1.06	0.84	0.96	0.90	0.68	0.72	0.88	0.69	0.80	0.74	0.75	0.07
186	Indirubin	1.14	0.97	1.16	1.42	0.97	0.81	1.08	0.79	0.82	0.76	0.80	0.83	0.82	0.73	0.76	0.70	0.74	0.77	0.76	0.74	0.02
187	Quercetin	1.05	0.82	0.92	0.91	1.09	0.92	0.95	0.73	0.59	0.64	0.65	0.54	0.73	0.77	0.62	0.67	0.69	0.57	0.77	0.68	0.08
188	Chrysophanic Acid	0.91	1.26	1.12	0.98	1.35	0.64	1.04	1.10	1.18	1.31	1.00	0.98	1.39	1.06	1.13	1.26	0.96	0.94	1.33	1.11	0.16
189	Imatinib (STI571)	0.42	0.37	0.33	0.34	0.33	0.30	0.35	0.67	0.58	0.54	0.53	0.67	0.65	1.92	1.66	1.55	1.51	1.93	1.88	1.74	0.19
190	Phenformin HCl	1.33	0.96	1.18	1.54	1.26	1.03	1.22	1.02	1.03	1.23	0.97	1.08	1.32	0.84	0.85	1.01	0.80	0.89	1.08	0.91	0.11
191	TAK-733	0.23	0.22	0.26	0.21	0.22	0.19	0.22	0.53	0.44	0.50	0.44	0.43	0.44	2.40	2.00	2.24	1.98	1.92	1.99	2.09	0.19
192	AZD5438	0.21	0.21	0.23	0.21	0.23	0.21	0.22	0.46	0.42	0.49	0.53	0.50	0.74	2.12	1.93	2.28	2.45	2.30	3.42	2.42	0.52
193	PP121	0.40	0.39	0.36	0.34	0.42	0.45	0.39	0.45	0.39	0.46	0.47	0.45	0.58	1.15	0.99	1.17	1.21	1.15	1.48	1.19	0.16
194	OSI-027	0.48	0.58	0.70	0.41	0.66	0.52	0.56	0.56	0.56	0.44	0.60	0.51	0.48	1.00	0.99	0.78	1.08	0.91	0.86	0.94	0.11
195	Fostamatinib (R788)	0.22	0.39	0.32	0.34	0.31	0.28	0.31	0.40	0.39	0.42	0.41	0.35	0.55	1.30	1.26	1.36	1.33	1.13	1.76	1.36	0.21
196	LY2603618	0.89	0.66	0.72	0.65	0.65	0.91	0.75	0.77	0.89	0.98	0.76	1.03	0.76	1.03	1.19	1.31	1.01	1.38	1.01	1.16	0.16
197	PF-05212384 (PKI-587)	0.33	0.38	0.41	0.40	0.44	0.33	0.38	0.43	0.35	0.48	0.38	0.38	0.45	1.13	0.92	1.26	1.00	1.00	1.17	1.08	0.13
198	DCC-2036 (Rebastinib)	0.41	0.41	0.43	0.37	0.32	0.34	0.38	0.42	0.46	0.44	0.42	0.56	0.53	1.12	1.21	1.16	1.10	1.48	1.40	1.24	0.16
199	CCT128930	0.39	0.39	0.46	0.44	0.46	0.49	0.44	0.56	0.68	0.56	0.65	0.49	0.59	1.29	1.55	1.27	1.48	1.11	1.34	1.34	0.16
200	A66	0.33	0.57	0.57	0.89	0.71	1.14	0.70	0.44	0.61	0.67	0.58	0.85	0.84	0.63	0.87	0.95	0.82	1.20	1.20	0.95	0.22
201	NU7441 (KU-57788)	0.43	0.68	0.57	0.55	0.52	0.55	0.55	0.55	0.58	0.52	0.51	0.54	0.51	1.00	1.06	0.94	0.93	0.98	0.92	0.97	0.05
202	GSK2126458 (GSK458)	0.29	0.30	0.24	0.27	0.32	0.28	0.28	0.35	0.40	0.32	0.37	0.52	0.41	1.23	1.41	1.13	1.32	1.85	1.45	1.40	0.25
203	WYE-125132 (WYE-132)	0.38	0.44	0.45	0.45	0.42	0.39	0.42	0.50	0.48	0.50	0.45	0.51	0.48	1.18	1.15	1.20	1.08	1.21	1.15	1.16	0.05
204	A-674563	0.24	0.23	0.26	0.27	0.16	0.24	0.23	0.48	0.45	0.37	0.47	0.38	0.59	2.04	1.92	1.57	2.01	1.62	2.51	1.94	0.34
205	AS-252424	1.14	0.98	1.08	0.55	1.16	1.55	1.08	0.56	0.65	0.73	0.44	1.20	0.70	0.52	0.60	0.67	0.41	1.12	0.65	0.66	0.24
206	PF-00562271	0.70	0.71	0.58	0.77	0.47	0.60	0.64	0.33	0.43	0.38	0.37	0.41	0.44	0.52	0.68	0.59	0.58	0.64	0.69	0.62	0.06
207	Trametinib (GSK1120212)	0.29	0.34	0.35	0.38	0.43	0.35	0.36	0.29	0.28	0.29	0.35	0.39	0.37	0.82	0.79	0.82	0.97	1.08	1.04	0.92	0.13
208	Flavopiridol HCl	0.38	0.38	0.35	0.29	0.29	0.32	0.33	0.32	0.29	0.37	0.36	0.36	0.33	0.96	0.88	1.11	1.08	1.08	1.00	1.02	0.09
209	Ibrutinib (PCI-32765)	0.63	0.71	0.77	0.77	0.49	0.80	0.69	0.44	0.45	0.47	0.48	0.37	0.45	0.63	0.64	0.67	0.70	0.53	0.64	0.64	0.06
210	AS-604850	0.27	0.58	0.63	0.48	0.50	0.43	0.48	0.27	0.33	0.39	0.32	0.29	0.31	0.56	0.69	0.82	0.66	0.60	0.64	0.66	0.09
211	CAY10505	0.84	0.70	0.91	0.79	0.77	0.85	0.81	0.42	0.41	0.55	0.47	0.48	0.41	0.51	0.51	0.68	0.58	0.59	0.51	0.56	0.07
212	CHIR-124	0.27	0.24	0.27	0.20	0.25	0.28	0.25	0.39	0.34	0.32	0.38	0.31	0.35	1.52	1.34	1.24	1.49	1.22	1.38	1.37	0.12
213	NVP-BSK805 2HCl	0.35	0.28	0.29	0.31	0.38	0.31	0.32	0.44	0.34	0.32	0.38	0.32	0.34	1.37	1.07	1.00	1.18	0.99	1.06	1.11	0.14
214	R547	0.29	0.29	0.27	0.25	0.23	0.24	0.26	0.30	0.26	0.26	0.25	0.26	0.28	1.13	1.00	1.00	0.97	1.01	1.07	1.03	0.06
215	WAY-600	0.30	0.31	0.32	0.29	0.26	0.30	0.30	0.40	0.33	0.34	0.32	0.34	0.31	1.35	1.12	1.14	1.10	1.14	1.06	1.15	0.10
216	TG101209	0.23	0.28	0.23	0.24	0.23	0.25	0.24	0.27	0.24	0.27	0.26	0.26	0.34	1.12	0.96	1.10	1.06	1.07	1.40	1.12	0.15
217	GDC-0980 (RG7422)	0.48	0.50	0.39	0.40	0.46	0.44	0.45	0.36	0.38	0.51	0.39	0.66	0.47	0.82	0.85	1.14	0.88	1.48	1.05	1.04	0.25
218	A-769662	1.20	1.18	1.08	1.39	1.27	1.08	1.20	1.34	1.27	1.21	0.97	1.08	1.32	1.12	1.06	1.01	0.81	0.90	1.10	1.00	0.12
219	CH5132799	0.77	0.72	0.50	0.66	0.53	0.53	0.62	0.46	0.43	0.63	0.51	0.55	0.48	0.75	0.71	1.03	0.83	0.89	0.78	0.83	0.12
220	KX2-391	0.50	0.47	0.47	0.46	0.40	0.61	0.48	0.52	0.56	0.52	0.64	0.53	0.52	1.08	1.15	1.08	1.33	1.10	1.07	1.14	0.10
221	GSK1838705A	1.00	1.01	1.36	1.02	0.98	0.93	1.05	0.50	0.53	0.56	0.57	0.59	0.61	0.48	0.51	0.53	0.54	0.57	0.58	0.53	0.04
222	TAK-901	0.23	0.26	0.30	0.24	0.27	0.30	0.27	0.55	0.45	0.50	0.41	0.38	0.48	2.04	1.66	1.86	1.51	1.42	1.80	1.72	0.23
223	AMG-900	0.83	0.84	0.96	0.67	1.16	0.90	0.89	0.70	0.63	0.79	0.64	0.78	0.90	0.78	0.70	0.89	0.72	0.88	1.01	0.83	0.12
224	ZM 336372	0.61	0.41	0.49	0.45	0.40	0.46	0.47	0.52	0.59	0.55	0.67	0.46	0.60	1.10	1.24	1.17	1.42	0.98	1.26	1.19	0.15
225	PH-797804	0.76	0.66	0.76	0.76	0.55	0.57	0.68	0.71	0.79	0.80	0.72	0.72	0.81	1.05	1.17	1.18	1.07	1.07	1.20	1.12	0.07
226	Dacomitinib (PF299804, PF299)	0.28	0.23	0.23	0.21	0.25	0.29	0.25	0.40	0.40	0.38	0.49	0.46	0.45	1.61	1.63	1.54	1.96	1.84	1.83	1.73	0.16
227	AG-1478 (Tyrphostin AG-1478)	0.85	1.30	0.89	0.95	0.75	0.65	0.90	0.93	0.91	1.12	1.08	0.89	0.88	1.03	1.01	1.25	1.20	0.99	0.98	1.08	0.12

228	***SB415286***	1.20	1.12	1.25	0.99	1.23	0.94	1.12	0.88	0.89	0.72	0.81	0.90	0.92	0.78	0.79	0.64	0.72	0.81	0.82	0.76	0.07
229	***Crenolanib (CP-868596)***	0.38	0.36	0.31	0.33	0.32	0.38	0.35	0.46	0.49	0.54	0.43	0.55	0.50	1.32	1.41	1.55	1.24	1.58	1.46	1.43	0.13
230	***MK-8776 (SCH 900776)***	0.63	0.74	0.70	0.65	0.51	0.41	0.61	0.48	0.52	0.58	0.63	0.57	0.86	0.79	0.86	0.97	1.05	0.93	1.42	1.00	0.22
231	***TG101348 (SAR302503)***	0.41	0.35	0.37	0.39	0.39	0.38	0.38	0.39	0.42	0.37	0.43	0.50	0.42	1.04	1.09	0.96	1.13	1.30	1.09	1.10	0.11
232	***GSK1070916***	0.52	0.72	0.60	0.40	0.40	0.30	0.49	0.57	0.47	0.46	0.51	0.35	0.45	1.16	0.95	0.93	1.04	0.72	0.92	0.95	0.15
233	***PHA-767491***	0.46	0.43	0.45	0.39	0.36	0.36	0.41	0.38	0.35	0.39	0.37	0.52	0.63	0.94	0.85	0.95	0.91	1.27	1.53	1.08	0.27
234	***PF-04691502***	0.61	0.39	0.65	0.38	0.41	0.41	0.48	0.60	0.41	0.39	0.43	0.42	0.51	1.27	0.86	0.81	0.91	0.89	1.08	0.97	0.17
235	***CCT137690***	0.42	0.39	0.37	0.34	0.36	0.23	0.35	0.40	0.35	0.42	0.30	0.37	0.60	1.14	1.00	1.19	0.84	1.04	1.69	1.15	0.29
236	***CHIR-98014***	0.50	0.56	0.37	0.38	0.40	0.42	0.44	0.52	0.45	0.34	0.40	0.42	0.65	1.19	1.04	0.77	0.92	0.97	1.47	1.06	0.25
237	***AZ 628***	0.50	0.40	0.45	0.44	0.38	0.36	0.42	0.51	0.47	0.40	0.44	0.44	0.47	1.22	1.11	0.95	1.04	1.05	1.12	1.08	0.09
238	***AMG-458***	0.77	0.67	0.48	0.54	0.63	0.49	0.60	0.70	0.51	0.56	0.54	0.46	0.62	1.17	0.85	0.95	0.90	0.77	1.04	0.94	0.14
239	***BGT226 (NVP-BGT226)***	0.32	0.32	0.32	0.35	0.28	0.29	0.31	0.54	0.45	0.57	0.54	0.48	0.43	1.73	1.45	1.85	1.74	1.54	1.39	1.62	0.18
240	***Milciclib (PHA-848125)***	0.31	0.35	0.32	0.35	0.30	0.25	0.31	0.36	0.33	0.40	0.39	0.35	0.46	1.14	1.05	1.27	1.26	1.12	1.46	1.21	0.14
241	***HER2-Inhibitor-1***	1.37	1.25	1.14	1.47	0.91	1.11	1.21	1.09	0.98	0.97	0.92	0.79	0.77	0.90	0.81	0.81	0.76	0.66	0.63	0.76	0.10
242	***Varlitinib***	1.20	1.03	0.89	1.11	0.90	0.81	0.99	0.58	0.63	0.61	0.74	0.72	0.80	0.59	0.63	0.61	0.75	0.73	0.80	0.69	0.09
243	***Wortmannin***	1.24	1.04	1.30	1.59	1.30	0.99	1.24	1.49	1.47	1.18	1.13	1.17	0.92	1.20	1.18	0.95	0.91	0.94	0.74	0.99	0.18
244	***CUDC-907***	0.32	0.34	0.32	0.31	0.27	0.24	0.30	0.45	0.40	0.40	0.50	0.42	0.44	1.49	1.33	1.33	1.67	1.39	1.46	1.45	0.13
245	***NVP-BVU972***	1.34	1.36	1.54	1.59	1.36	1.16	1.39	1.10	1.00	1.29	1.15	1.03	1.04	0.79	0.72	0.93	0.83	0.74	0.75	0.79	0.08
246	***Alectinib (CH5424802)***	0.40	0.36	0.28	0.30	0.30	0.39	0.34	0.55	0.45	0.44	0.48	0.44	0.54	1.62	1.33	1.30	1.41	1.29	1.59	1.42	0.15
247	***3-Methyladenine***	1.09	1.21	1.17	1.22	1.19	1.25	1.19	1.16	0.95	0.95	0.83	0.66	0.33	0.98	0.80	0.80	0.70	0.55	0.28	0.68	0.24
248	***Dinaciclib (SCH727965)***	0.29	0.33	0.27	0.26	0.26	0.27	0.28	0.37	0.47	0.33	0.47	0.42	0.51	1.30	1.68	1.18	1.66	1.51	1.81	1.52	0.24
249	***Dovitinib (TKI-258) Dilactic Acid***	0.22	0.23	0.22	0.25	0.24	0.25	0.23	0.39	0.35	0.37	0.42	0.41	0.44	1.68	1.51	1.58	1.80	1.76	1.87	1.70	0.14
250	***MK-5108 (VX-689)***	0.30	0.30	0.25	0.25	0.26	0.24	0.27	0.32	0.37	0.36	0.41	0.39	0.46	1.20	1.39	1.34	1.54	1.47	1.72	1.44	0.18
251	***MK-2461***	0.32	0.36	0.37	0.35	0.37	0.29	0.34	0.49	0.41	0.42	0.43	0.39	0.38	1.44	1.19	1.24	1.26	1.13	1.10	1.22	0.12
252	***AZD2014***	0.36	0.27	0.33	0.30	0.31	0.29	0.31	0.43	0.40	0.46	0.48	0.49	0.54	1.38	1.31	1.49	1.53	1.58	1.73	1.50	0.15
253	***TAK-285***	1.04	1.11	0.78	0.97	0.76	1.00	0.95	1.32	1.11	0.99	0.92	0.87	0.91	1.40	1.17	1.05	0.97	0.92	0.97	1.08	0.18
254	***INCB28060***	1.16	1.04	1.35	1.08	1.07	1.18	1.14	1.08	1.30	1.09	1.28	1.05	1.28	0.94	1.13	0.95	1.12	0.92	1.12	1.03	0.10
255	***Tofacitinib (CP-690550,Tasocitinib)***	1.29	1.80	1.32	1.29	1.33	1.29	1.39	1.26	0.96	1.08	1.05	1.12	0.99	0.91	0.69	0.78	0.76	0.81	0.72	0.78	0.08
256	***Sotrastaurin***	0.63	0.89	0.91	1.13	0.70	0.91	0.86	0.73	0.87	0.76	0.71	0.72	1.19	0.84	1.01	0.88	0.83	0.84	1.39	0.96	0.22
257	***WP1066***	1.46	1.45	1.37	1.03	1.05	1.00	1.23	1.53	1.39	1.10	1.00	1.12	0.92	1.24	1.13	0.89	0.82	0.92	0.75	0.96	0.19
258	***AZD4547***	0.31	0.30	0.25	0.25	0.27	0.26	0.27	0.55	0.48	0.47	0.65	0.63	0.69	2.02	1.74	1.73	2.37	2.28	2.53	2.11	0.34
259	***CEP-33779***	0.35	0.41	0.46	0.51	0.46	0.32	0.42	0.65	0.59	0.54	0.47	0.50	0.45	1.55	1.42	1.28	1.12	1.18	1.07	1.27	0.18
260	***Dabrafenib (GSK2118436)***	0.66	0.46	0.57	0.64	0.40	0.70	0.57	0.49	0.47	0.40	0.57	0.59	1.14	0.86	0.81	0.70	0.99	1.04	1.99	1.07	0.47
261	***GDC-0068***	0.35	0.45	0.43	0.57	0.40	0.43	0.44	0.72	0.75	0.59	0.55	0.60	0.56	1.63	1.70	1.35	1.25	1.36	1.27	1.43	0.19
262	***INK 128 (MLN0128)***	0.41	0.41	0.48	0.49	0.36	0.38	0.42	0.54	0.47	0.52	0.51	0.61	0.61	1.28	1.12	1.24	1.21	1.45	1.45	1.29	0.13
263	***BYL719***	0.73	0.80	0.87	0.84	0.55	0.57	0.73	0.92	0.97	0.80	0.73	0.72	0.67	1.27	1.33	1.10	1.00	1.00	0.92	1.10	0.16
264	***Tyrphostin AG 879***	0.47	0.41	0.34	0.37	0.35	0.35	0.38	0.53	0.52	0.49	0.52	0.62	0.75	1.40	1.35	1.29	1.36	1.61	1.97	1.50	0.26
265	***Torin 2***	0.28	0.22	0.20	0.19	0.23	0.22	0.22	0.55	0.48	0.44	0.38	0.43	0.38	2.45	2.17	1.99	1.69	1.93	1.71	1.99	0.29
266	***TAE226 (NVP-TAE226)***	0.65	0.48	0.63	0.56	0.48	0.58	0.56	0.40	0.39	0.47	0.43	0.42	0.43	0.71	0.69	0.84	0.77	0.74	0.76	0.75	0.05
267	***Tideglusib***	0.82	0.64	0.74	0.59	0.53	1.01	0.72	0.61	0.59	0.42	0.44	0.46	0.41	0.84	0.82	0.59	0.61	0.64	0.57	0.68	0.12
268	***TPCA-1***	0.72	0.89	0.63	0.64	0.51	0.55	0.66	0.47	0.40	0.50	0.51	0.57	0.50	0.72	0.61	0.77	0.77	0.87	0.76	0.75	0.09
269	***Torin 1***	0.48	0.41	0.32	0.34	0.24	0.32	0.35	0.57	0.42	0.41	0.38	0.48	0.42	1.62	1.20	1.17	1.08	1.38	1.20	1.28	0.20
270	***SAR131675***	0.58	0.48	0.49	0.35	0.47	0.43	0.47	0.45	0.48	0.44	0.40	0.41	0.60	0.97	1.03	0.94	0.85	0.87	1.28	0.99	0.16
271	***BI-D1870***	0.52	0.57	0.54	0.60	0.52	0.65	0.57	0.51	0.46	0.39	0.39	0.39	0.34	0.89	0.81	0.69	0.69	0.70	0.59	0.73	0.11
272	***Semaxanib (SU5416)***	0.33	0.44	0.52	0.36	0.39	0.35	0.40	0.37	0.41	0.43	0.42	0.47	0.50	0.93	1.04	1.08	1.07	1.17	1.25	1.09	0.11
273	***Golvatinib (E7050)***	0.36	0.34	0.32	0.29	0.26	0.25	0.30	0.59	0.48	0.42	0.46	0.49	0.47	1.94	1.59	1.37	1.52	1.60	1.53	1.59	0.19

274	*IMD 0354*	0.26	0.22	0.19	0.21	0.20	0.21	0.22	0.42	0.44	0.49	0.39	0.46	0.57	1.93	2.01	2.24	1.79	2.12	2.61	2.11	0.29
275	*WHI-P154*	0.71	0.67	0.47	0.39	0.46	0.29	0.50	0.74	0.69	0.63	0.51	0.45	0.52	1.47	1.38	1.25	1.03	0.89	1.03	1.18	0.23
276	*TG100713*	0.30	0.34	0.40	0.46	0.39	0.38	0.38	0.49	0.40	0.49	0.48	0.44	0.52	1.28	1.06	1.29	1.25	1.15	1.36	1.23	0.11
277	*GW5074*	1.07	1.02	1.02	1.13	1.04	0.96	1.04	1.46	1.18	1.32	1.28	1.18	1.31	1.40	1.13	1.27	1.23	1.13	1.26	1.24	0.10
278	*IKK-16 (IKK Inhibitor VII)*	0.30	0.31	0.22	0.27	0.24	0.21	0.26	0.59	0.60	0.47	0.87	0.40	0.72	2.31	2.33	1.84	3.38	1.57	2.79	2.37	0.65
279	*PF-562271*	0.71	0.59	0.74	0.51	0.65	0.47	0.61	0.68	0.59	0.56	0.62	0.51	0.50	1.11	0.96	0.92	1.01	0.84	0.82	0.94	0.11
280	*NU7026*	0.69	0.90	0.74	1.13	0.85	0.80	0.85	1.00	1.14	0.96	1.05	0.95	1.21	1.17	1.34	1.13	1.23	1.12	1.41	1.23	0.12
281	*Tyrphostin 9*	0.44	0.67	0.37	0.30	0.28	0.27	0.39	0.61	0.70	0.39	0.41	0.47	0.41	1.58	1.80	1.01	1.05	1.21	1.06	1.28	0.33
282	*ZM 323881 HCl*	0.60	0.79	0.79	0.70	0.74	0.73	0.73	0.74	0.68	0.66	0.73	0.85	0.99	1.02	0.94	0.91	1.01	1.17	1.36	1.07	0.17
283	*ZM 306416*	0.85	0.88	1.05	1.36	1.07	1.43	1.11	1.11	1.00	0.98	0.88	1.04	0.73	1.00	0.91	0.88	0.79	0.94	0.66	0.86	0.12
284	*GNF-2*	1.04	1.33	0.76	1.34	1.24	1.11	1.14	1.50	1.64	1.35	1.46	1.62	1.83	1.32	1.44	1.19	1.28	1.43	1.61	1.38	0.15
285	*S-Ruxolitinib (INCB018424)*	0.85	1.23	1.13	1.06	1.08	1.04	1.07	1.03	0.96	0.97	0.84	0.91	0.78	0.96	0.90	0.91	0.79	0.85	0.73	0.86	0.09
286	*PF-477736*	0.23	0.27	0.20	0.27	0.24	0.25	0.24	0.38	0.35	0.39	0.40	0.39	0.47	1.59	1.44	1.61	1.66	1.61	1.94	1.64	0.16
287	*Go 6983*	0.28	0.24	0.23	0.26	0.23	0.23	0.24	0.61	0.50	0.45	0.41	0.36	0.47	2.49	2.05	1.84	1.69	1.47	1.93	1.91	0.35
288	*BAY 11-7082*	0.35	0.46	0.40	0.60	0.46	0.40	0.45	0.74	0.79	0.90	0.84	0.85	1.31	1.67	1.76	2.01	1.88	1.90	2.93	2.03	0.46
289	*Icotinib*	1.35	1.21	1.12	0.97	1.33	1.08	1.18	1.20	1.79	1.54	1.79	1.72	1.88	1.02	1.52	1.31	1.52	1.47	1.60	1.41	0.21
290	*CHIR-99021 (CT99021) HCl*	1.35	1.22	1.40	1.43	1.35	0.96	1.29	1.37	1.42	1.03	0.84	1.14	0.78	1.06	1.10	0.80	0.65	0.89	0.61	0.85	0.21
291	*TAK-715*	1.61	1.87	1.58	1.56	1.30	1.52	1.57	1.54	1.85	1.58	1.61	1.54	1.46	0.98	1.18	1.01	1.02	0.98	0.93	1.02	0.09
292	*Pazopanib*	0.72	0.46	0.48	0.49	0.40	0.51	0.51	0.52	0.52	0.34	0.51	0.59	0.51	1.03	1.02	0.66	0.99	1.17	0.99	0.98	0.17
293	*Piceatannol*	1.51	1.69	1.79	1.09	1.17	1.04	1.38	0.95	1.54	1.16	0.73	0.70	0.61	0.69	1.11	0.84	0.53	0.51	0.44	0.69	0.25
294	*SC-514*	0.93	1.09	1.20	0.74	0.71	0.79	0.91	1.19	0.91	1.15	0.61	0.54	0.48	1.31	1.00	1.26	0.67	0.59	0.52	0.89	0.35
295	*Tofacitinib (CP-690550) Citrate*	1.93	1.43	1.71	1.53	1.23	1.14	1.49	1.20	1.21	1.20	1.12	1.38	1.22	0.81	0.81	0.80	0.75	0.92	0.81	0.82	0.06
296	*Fingolimod (FTY720) HCl*	0.29	0.28	0.22	0.28	0.25	0.25	0.26	0.33	0.40	0.28	0.31	0.29	0.30	1.27	1.54	1.06	1.19	1.10	1.15	1.22	0.17
297	*VX-702*	2.05	1.71	1.86	2.41	2.53	2.29	2.14	1.71	1.39	1.58	1.60	1.63	1.37	0.80	0.65	0.74	0.75	0.76	0.64	0.72	0.06
298	*AP26113*	0.36	0.31	0.31	0.26	0.23	0.25	0.29	0.26	0.28	0.24	0.41	0.41	0.33	0.91	0.99	0.85	1.45	1.42	1.17	1.13	0.26
299	*MEK162 (ARRY-162, ARRY-438162)*	0.60	0.51	0.52	0.44	0.38	0.36	0.47	0.60	0.49	0.41	0.41	0.45	0.45	1.27	1.05	0.87	0.87	0.95	0.95	1.00	0.15
300	*PP2*	0.53	0.45	0.27	0.34	0.32	0.31	0.37	0.53	0.50	0.57	0.56	0.42	0.45	1.43	1.34	1.53	1.51	1.13	1.22	1.36	0.16
301	*CZC24832*	1.26	1.08	1.03	0.95	1.02	0.98	1.06	1.08	0.99	1.10	1.41	1.29	1.13	1.03	0.94	1.04	1.34	1.23	1.07	1.11	0.15
302	*IPI-145 (INK1197)*	0.96	0.87	0.81	0.84	0.67	0.50	0.77	0.72	0.67	0.69	0.80	0.76	0.72	0.93	0.87	0.89	1.03	0.99	0.93	0.94	0.06
303	*XL388*	0.83	0.56	0.50	0.70	0.48	0.44	0.59	0.88	0.87	0.77	0.81	0.66	0.61	1.50	1.48	1.31	1.38	1.13	1.04	1.31	0.19
304	*XL019*	0.83	0.74	0.70	0.56	0.42	0.30	0.59	0.39	0.47	0.43	0.39	0.38	0.49	0.66	0.79	0.72	0.66	0.64	0.82	0.72	0.08
305	*PD168393*	0.75	0.79	0.61	0.71	0.61	0.49	0.66	0.74	0.52	0.63	0.63	0.53	0.50	1.13	0.79	0.96	0.96	0.80	0.76	0.90	0.14
306	*AZ20*	0.43	0.38	0.47	0.34	0.32	0.30	0.37	0.69	0.55	0.54	0.63	0.49	0.60	1.84	1.48	1.44	1.68	1.31	1.60	1.56	0.19
307	*PP1*	0.46	0.45	0.32	0.31	0.32	0.32	0.36	0.69	0.64	0.46	0.53	0.60	0.42	1.89	1.74	1.26	1.46	1.64	1.15	1.52	0.29
308	*MK-8745*	0.63	0.63	0.69	0.48	0.49	0.62	0.59	0.64	0.75	0.80	0.77	0.77	0.97	1.08	1.27	1.35	1.30	1.29	1.64	1.32	0.18
309	*LDK378*	1.08	0.27	0.25	0.19	0.21	0.23	0.37	1.07	0.53	0.49	0.59	0.56	0.46	2.89	1.41	1.32	1.58	1.52	1.23	1.66	0.62
310	*IPA-3*	0.90	0.83	0.69	1.09	0.55	0.46	0.75	0.38	0.41	0.37	0.38	0.38	0.44	0.50	0.55	0.50	0.50	0.51	0.59	0.52	0.04
311	*VE-822*	0.71	0.57	0.57	0.38	0.42	0.50	0.53	0.93	0.72	0.85	0.77	0.79	0.62	1.76	1.37	1.62	1.47	1.50	1.18	1.48	0.20
312	*AZD3463*	0.19	0.15	0.17	0.18	0.13	0.17	0.17	0.54	0.56	0.53	0.57	0.52	0.64	3.20	3.37	3.18	3.42	3.08	3.83	3.35	0.27
313	*NU6027*	0.77	1.23	0.99	0.77	0.93	0.75	0.91	0.76	0.47	0.59	0.64	0.57	0.48	0.84	0.52	0.65	0.70	0.63	0.53	0.65	0.12
314	*TIC10*	1.13	1.05	0.96	1.14	1.26	1.50	1.17	1.29	1.25	1.42	1.36	1.08	1.32	1.10	1.07	1.21	1.16	0.92	1.12	1.10	0.10
315	*CGK 733*	1.26	1.34	1.36	1.13	1.32	0.79	1.20	1.23	1.04	0.84	0.96	0.94	0.65	1.02	0.86	0.70	0.79	0.78	0.54	0.78	0.16
316	*AZD1080*	0.98	0.87	1.04	0.89	0.92	0.67	0.89	0.71	0.77	0.65	0.87	0.81	0.91	0.79	0.86	0.73	0.98	0.91	1.01	0.88	0.11
317	*10058-F4*	1.41	1.34	0.97	1.18	1.23	1.00	1.19	1.41	1.17	1.43	1.42	1.24	0.81	1.18	0.98	1.20	1.19	1.04	0.69	1.05	0.20
318	*LY2835219*	0.63	0.19	0.22	0.24	0.23	0.18	0.28	0.40	0.35	0.65	0.68	0.60	0.72	1.43	1.25	2.30	2.41	2.12	2.56	2.01	0.54
319	*SSR128129E*	1.54	1.56	1.46	1.96	1.37	0.98	1.48	1.41	1.12	1.02	1.24	1.09	0.62	0.95	0.76	0.69	0.84	0.74	0.42	0.73	0.18

320	*AVL-292*	0.46	0.40	0.28	0.32	0.25	0.21	0.32	0.44	0.41	0.40	0.55	0.53	0.63	1.38	1.30	1.24	1.73	1.66	1.97	1.55	0.29
321	*SKI II*	1.11	1.18	1.26	1.15	1.18	0.87	1.12	0.81	0.74	0.67	0.58	0.61	0.60	0.72	0.66	0.59	0.51	0.54	0.54	0.59	0.08
322	*GZD824*	0.19	0.19	0.20	0.22	0.19	0.24	0.21	0.62	0.39	0.39	0.45	0.39	0.52	2.97	1.90	1.87	2.18	1.89	2.50	2.22	0.44
323	*RKI-1447*	0.84	0.57	0.67	0.55	0.37	0.46	0.58	1.02	0.81	0.80	0.73	0.58	0.56	1.77	1.41	1.39	1.27	1.00	0.97	1.30	0.29
324	*BIO*	0.22	0.16	0.21	0.15	0.18	0.32	0.21	0.47	0.50	0.44	0.46	0.45	0.63	2.28	2.43	2.12	2.22	2.21	3.04	2.38	0.34
325	*Ro 31-8220 Mesylate*	0.31	0.25	0.27	0.31	0.27	0.27	0.28	0.50	0.39	0.45	0.50	0.47	0.44	1.78	1.39	1.60	1.78	1.68	1.57	1.63	0.15
326	*Skepinone-L*	1.02	2.10	1.65	2.47	1.12	1.48	1.64	2.33	2.51	2.24	1.90	1.51	1.72	1.42	1.53	1.36	1.16	0.92	1.05	1.24	0.24
327	*AZD2858*	0.66	0.63	0.82	0.80	0.77	0.59	0.71	0.65	0.74	0.63	0.88	0.88	0.87	0.92	1.04	0.89	1.23	1.23	1.23	1.09	0.16
328	*CNX-774*	0.97	0.89	1.18	1.09	1.00	1.17	1.05	0.78	0.69	0.70	0.87	0.90	0.78	0.74	0.66	0.66	0.83	0.86	0.74	0.75	0.08
329	*CO-1686 (AVL-301)*	1.14	0.95	0.80	0.71	0.89	0.85	0.89	0.59	0.61	0.51	0.57	0.51	0.54	0.66	0.69	0.57	0.64	0.58	0.60	0.62	0.05
330	*TAK-632*	0.48	0.62	0.39	0.53	0.45	0.51	0.50	0.38	0.42	0.37	0.52	0.45	0.55	0.76	0.85	0.75	1.04	0.90	1.10	0.90	0.15
331	*ZCL278*	1.57	1.68	1.60	1.18	1.13	1.67	1.47	1.42	1.42	1.56	1.66	1.63	1.90	0.97	0.96	1.06	1.13	1.11	1.30	1.09	0.12
332	*WZ4003*	1.64	0.48	0.47	0.54	0.46	0.50	0.68	0.74	0.61	0.67	0.61	0.58	0.72	1.09	0.89	0.99	0.90	0.85	1.06	0.96	0.10
333	*EHop-016*	0.27	1.19	0.24	0.25	0.29	0.27	0.42	0.66	0.97	0.64	0.81	0.94	0.74	1.57	2.33	1.53	1.93	2.24	1.78	1.90	0.34
334	*TG003*	1.25	1.82	1.35	1.69	1.80	2.02	1.66	1.46	1.18	1.09	1.16	1.22	1.22	0.88	0.72	0.66	0.70	0.74	0.74	0.74	0.08
335	*Sorafenib*	0.50	0.38	0.43	0.52	0.47	0.47	0.46	0.70	0.57	0.72	0.72	0.60	0.59	1.51	1.23	1.55	1.55	1.30	1.27	1.40	0.15
336	*AR-A014418*	1.68	1.37	1.45	1.75	1.67	1.29	1.54	1.57	0.95	1.14	1.13	1.04	1.34	1.02	0.62	0.74	0.74	0.67	0.87	0.78	0.15
337	*GSK2636771*	1.27	0.94	0.79	1.07	0.91	1.32	1.05	1.00	0.88	1.26	1.03	0.93	0.77	0.95	0.84	1.20	0.98	0.89	0.73	0.93	0.16
338	*PQ 401*	1.15	1.49	1.35	1.12	1.39	1.04	1.26	1.30	1.25	0.90	1.19	1.14	1.59	1.04	0.99	0.72	0.95	0.91	1.27	0.98	0.18
339	*ZM 39923 HCl*	1.32	1.07	1.40	0.94	1.13	0.85	1.12	1.13	1.59	0.91	1.08	1.23	1.76	1.02	1.42	0.81	0.97	1.10	1.57	1.15	0.29
340	*SMI-4a*	1.36	1.65	1.29	1.26	1.05	1.13	1.29	1.52	1.24	1.50	1.12	1.26	1.39	1.18	0.96	1.17	0.87	0.98	1.08	1.04	0.12
341	*VE-821*	0.65	0.94	0.74	0.76	0.73	0.78	0.77	1.20	1.14	1.11	1.13	1.39	1.27	1.56	1.49	1.45	1.47	1.81	1.66	1.57	0.14
342	*AG-18*	1.39	0.90	1.01	1.09	1.38	1.42	1.20	1.16	1.04	1.07	1.15	0.99	1.17	0.97	0.87	0.89	0.96	0.82	0.97	0.91	0.06
343	*CEP-32496*	0.33	0.32	0.26	0.38	0.37	0.37	0.34	0.47	0.46	0.43	0.35	0.41	0.43	1.39	1.36	1.27	1.03	1.21	1.27	1.25	0.13
344	*AZD5363*	0.71	0.69	0.49	0.50	0.42	0.62	0.57	0.51	0.48	0.51	0.62	0.60	0.61	0.89	0.85	0.89	1.09	1.05	1.07	0.97	0.11
345	*TCS 359*	1.50	1.28	1.52	1.06	1.15	1.34	1.31	1.72	1.63	1.61	2.09	1.79	0.81	1.31	1.25	1.24	1.60	1.37	0.62	1.23	0.33
346	*Tyrphostin AG 1296*	1.00	1.06	1.16	0.78	1.36	0.27	0.94	1.29	1.55	1.63	1.41	1.31	0.60	1.38	1.66	1.74	1.51	1.39	0.64	1.39	0.39
347	*NSC 23766*	1.01	1.00	0.84	0.64	0.70	1.46	0.94	0.90	0.96	0.86	0.99	0.85	1.03	0.96	1.02	0.91	1.05	0.90	1.09	0.99	0.08
348	*PRT062607 (P505-15, BIIB057) HCl*	0.27	0.24	0.24	0.24	0.22	0.25	0.24	0.46	0.47	0.47	0.53	0.44	0.54	1.92	1.95	1.95	2.19	1.84	2.24	2.02	0.16
349	*Butein*	1.26	1.34	1.30	1.38	1.64	1.14	1.34	1.06	1.19	1.06	0.99	0.93	0.64	0.79	0.88	0.79	0.74	0.69	0.47	0.73	0.14
350	*GDC-0349*	0.61	0.72	0.52	0.73	0.53	0.56	0.61	0.30	0.32	0.29	0.26	0.29	0.50	0.49	0.53	0.47	0.42	0.48	0.81	0.53	0.14
351	*BMS-345541*	0.96	0.75	0.28	0.36	0.32	0.41	0.51	0.55	0.73	0.27	0.29	0.22	0.28	1.08	1.43	0.53	0.56	0.43	0.55	0.76	0.40
352	*ETP-46464*	0.67	0.98	0.58	0.80	0.64	0.50	0.70	0.57	0.42	0.41	0.56	0.69	0.56	0.82	0.61	0.59	0.81	0.99	0.81	0.77	0.15
353	*Pacritinib (SB1518)*	0.54	0.32	0.36	0.29	0.32	0.25	0.35	0.32	0.30	0.37	0.30	0.42	0.51	0.94	0.88	1.07	0.88	1.23	1.47	1.08	0.23
354	*P276-00*	0.30	0.34	0.39	0.35	0.33	0.33	0.34	0.26	0.33	0.32	0.31	0.34	0.36	0.76	0.98	0.93	0.92	0.99	1.05	0.94	0.10
355	*Bardoxolone Methyl*	0.33	0.40	0.39	0.35	0.42	0.37	0.38	0.33	0.30	0.28	0.25	0.34	0.23	0.86	0.79	0.74	0.67	0.89	0.60	0.76	0.11
