# Supplementary material for: Chemical screening identifies ROCK as a target for recovering mitochondrial function in Hutchinson‐Gilford progeria syndrome
Source: Aging Cell. 2017 Mar 19;16(3):541–50. doi: 10.1111/acel.12584 (PMC5418208; doi:10.1111/acel.12584)
Supplement: Supplementary file 8 — Table S2 Detailed list of positive clones from yeast two‐hybrid screening. [file ACEL-16-541-s008.pdf]

Supplementary Table 2. Detailed list of positive clones from Yeast Two-Hybrid screening

BAIT: ROCK1-M2 (945-1113)

Hit	Gene	NCBI number	GAL4 AD-Fusion junction
**1**	Homo sapiens ras-related C3 botulinum toxin substrate 1 (rho family, small GTP binding protein Rac1) (RAC1), transcript variant Rac1b	NM_018890	In frame, Fused to 5' UTR of the gene

BAIT: ROCK2-M2 (976-1131)

Hit	Gene	NCBI number	GAL4 AD-Fusion junction
**1**	Homo sapiens pancreatic lipase-related protein 1 (PNLIPRP1), transcript variant 1	NM_006229	In frame, fused at 436/467 aa of PNLIPRP1
**4**	Homo sapiens ArfGAP with FG repeats 1 (AGFG1), transcript variant 3	NM_0011351 88	In frame, Fused to 5' UTR of the gene
**1**	Homo sapiens protein phosphatase 1, regulatory subunit 26 (PPP1R26)	NM_014811	In frame, fused at 837/1209 aa of PPP1R26
