# Supplementary material for: Chemical screening identifies ROCK as a target for recovering mitochondrial function in Hutchinson‐Gilford progeria syndrome
Source: Aging Cell. 2017 Mar 19;16(3):541–50. doi: 10.1111/acel.12584 (PMC5418208; doi:10.1111/acel.12584)
Supplement: Supplementary file 9 — Appendix S1 Experimental procedures. [file ACEL-16-541-s009.doc]

**Supporting Information**

**SI Experimental procedures**

**Yeast two-hybrid screen**

Yeast two-hybrid screening with GAL4 DNA-binding domain (BD)-fused ROCK1-M (residues 945–1113, 169 aa) and ROCK2-M (residues 976–1131, 156 aa) were performed using the human pancreas cDNA activation domain (AD) library as previously described . Briefly, the yeast strain PBN204 (Panbionet, Pohang, Korea) was co-transformed with two hybrid plasmids using the polyethylene glycol-lithium acetate method. In our screening, three reporter genes-URA3, ADE2, and lacZ, each under the control of different GAL4-binding sites-were used to minimize false positives. First, the transformants were spread on selection medium lacking leucine, tryptophan and uracil (SD-LWU). On this medium, the transformants grow when ROCK1-M or ROCK2-M interacts with the AD-prey proteins. Second, the positive colonies were grown on selective medium lacking leucine, tryptophan, and histidine (SD-LWA) in the presence of 2 mM 3-AT. Yeast transformants were selected on SD minimal medium lacking leucine and tryptophan (SD-LW). Transformants were replica plated onto various SD selection media, such as medium lacking leucine, tryptophan and uracil (SD-LWU), and medium lacking leucine, tryptophan and adenine (SD-LWA). Protein-protein interactions were tested using three independent reporters with different types of GAL4-binding sites. pGBKT7-polypyrimidine tract binding protein (PTB) and pGADT7-PTB served as the positive control for the protein-protein interaction . pGBKT7 (Clontech Laboratories, Inc., Mountain View, CA, USA) and pGADT7 (Clontech) were used as negative controls.

**RNA interference**

siRNAs targeting *ROCK1* (1130671; Bioneer, Daejeon, Korea), *ROCK2* (1130678; Bioneer), Rac1b (sense: GUU GGA GAA ACG UAC GGU AAG GAU A, anti-sense: U AUC CUU ACC GUA CGU UUC UCC AAC), and control siRNA (SS-1003) were synthesized by Bioneer (Daejeon, Korea). Cells were transfected with siRNA at a final concentration of 25 nM using Lipofectamine 2000 (Invitrogen).

**Plasmid construction**

Plasmid p3XFlag-Rac1b was constructed by inserting a cDNA encoding human Rac1b (GenBank accession NM_018890) into the p3XFLAG-CMV 10 vector (E7658-20UG; Sigma). Plasmid pcDNA3.1-Myc-Rac1b was constructed by inserting a cDNA encoding human Rac1b (GenBank accession NM_018890) into the pcDNA3.1-Myc-His A vector (V855-20; Invitrogen). Plasmid p3XFlag-ROCK1-M was constructed by inserting ROCK1-M (residues 945-1113, 169 aa) into the p3XFLAG-CMV 10 vector (E7658-20UG; Sigma).

***In vivo* co-immunoprecipitation**

The *in vivo* co-immunoprecipitation assay was performed as previously described. p3XFlag-ROCK1-M, and pcDNA3.1-Myc-Rac1b were transfected into HEK 293T cells. After 48 hr, the cells were washed with ice-cold phosphate-buffered saline (PBS) and lysed with lysis buffer [50 mM Tris-HCl (pH 7.4), 150 mM KCl, 1 mM PMSF, 2 mM benzamidine, 0.05% NP-40, protease inhibitor cocktail (04693116001; Roche, BASEL, Switzerland)], and sonicated using a Branson Sonifier 250. Insoluble material was removed by centrifugation at 10,000 × *g* for 10 min, and the supernatant was pre-cleared with protein G-agarose beads on a rotating wheel for 2 hr. The pre-cleared cell lysate was incubated with 5 μg high-affinity monoclonal anti-Flag antibody (F3165-.2MG; Sigma) for 2 hr and incubated with protein G-agarose beads overnight to immunoprecipitate the epitope-tagged proteins. As a control, 5 μg mouse IgG antibody (sc-2025; Santa Cruz Biotechnology) was used. The samples were washed twice with lysis buffer and twice with ice-cold PBS. Unless otherwise stated, all procedures were performed at 4°C. SDS sample buffer (20 μL) was added to the pellet, which was then heated to 95°C for 10 min. The proteins were separated by 10% SDS-PAGE and analyzed by immunoblotting.

**Subcellular fractionation**

Cells were homogenized by using a pre-chilled Dounce homogenizer in a detergent-free lysis buffer [10 mm Tris, pH 7.8, 0.2 mm EDTA, 320 mm sucrose, and protease inhibitor cocktail]. The homogenates were centrifuged at 2,000 × *g* for 8 min at 4°C and post nuclear supernatant (PNS) fractions were prepared. Supernatant fractions were collected by centrifugation at 12,000 × *g* for 20 min at 4°C. The pellets were resuspended in lysis buffer and centrifuged at 12,000 × *g* for 20 min to wash the remaining soluble fractions. Pellets (Mito-enriched; containing the mitochondrial fraction) and supernatants (SN; containing the cytosol fraction) were prepared at equal volumes.

**Immunofluorescence**

For immunofluorescence, cells were plated on Nunc Lab-Tek II Chamber Slides (154526; Thermo Fischer Scientific, Waltham, MA, USA), washed with ice-cold PBS, fixed with 4% paraformaldehyde/PBS for 15 min at room temperature, permeabilized with 0.1 % Triton X-100/PBS for 15 min, and blocked with 10% FBS in PBS for 1 hr. After incubation with rabbit anti-Rac1b (09-271; 1:100 dilution; Millipore), mouse anti-oxphos antibody (MS601; 1:100 dilution; MitoSciences), mouse anti-Lamin A/C antibody (MAB3211; 1:1,000 dilution; Millipore), and mouse anti- γH2AX antibody (560443; 1:100 dilution; BD Biosciences) overnight at 4°C, the cells were washed with ice-cold PBS three times and incubated with Cy3-conjugated anti-mouse antibodies (711-585-152; 1:400 dilution; Jackson Labs, West Grove, PA, USA), Cy5-conjugated anti-mouse antibodies (715-605-151; 1:400 dilution; Jackson Labs), or Cy3-conjugated anti-rabbit antibodies (711-165-152; 1:400 dilution; Jackson Labs) for 30 min at RT. The nuclei were then stained with DAPI (R37606; Invitrogen). Coverslips were washed with ice-cold PBS four times and then mounted on glass slides.

**Mass analysis**

To identify the Rac1b phosphorylation site, mass analysis was performed by Diatech Korea, Inc. HEK 293T cells were transiently transfected with Flag-Rab1b. To induce RhoA/ROCK activation, HEK 293T cells were treated with 0.5 μg/ml Rho Activator II (CN03-A; Cytoskeleton, DENVER, CO, USA). Whole-cell extracts were then immunoprecipitated with an anti-Flag antibody, resolved by SDS-PAGE, and in-gel-digested with trypsin. Peptides were analyzed by mass fingerprinting using MALDI-TOF-MS (Voyager-DE STR, Applied Biosystems, Foster City, CA, USA). The predicted peptides were identified using the Mascot Search Engine (www.matrixscience.com).

**Oxygen consumption rate (OCR), extracellular acidification rate (ECAR) analysis, and measurement of complex IV activity**

The XFe24 flux analyzer and a Prep Station (Seahorse Bioscience XFe24 Instrument, Billerica, MA, USA) were used according to the manufacturer’s protocol. Briefly, 5 × 10^4^ cells were distributed into each well of an XFe24 cell-culture plate from the XF24 FluxPak (100850-001; Seahorse Bioscience) and then cultured in a 5% CO_2_ incubator at 37°C for 16 hr. Next, the medium was replaced with XF Assay medium (102365-100; Seahorse Bioscience) supplemented with 25 mM glucose and 1 mM pyruvate for OCR or XF Base medium (102353-100; Seahorse Bioscience) supplemented with 2 mM L-Glutamine for ECAR. Cells were cultured for another 1 hr in a CO_2_-free incubator at 37°C. OCR was measured using an XF Cell Mito Stress Test Kit (101706-100; Seahorse Bioscience). ECAR was measured using an XF Glycolysis Stress Test kit (102194-100; Seahorse Bioscience). OCR is reported in pmoles/min and ECAR is reported in mpH/min. The aconitase inhibitor (deferiprone, 379409-5G; Sigma) was used as a metabolic shifter. For measurement of complex IV activity, cells were permeabilized with Mitochondrial Assay Solution (MAS) buffer (70 mM sucrose, 220 mM mannitol, 10 mM KH_2_PO_4_, 5 mM MgCl_2_, 2 mM HEPES, and 1 mM EGTA; pH 7.2) containing 2.5 nM XF Plasma membrane permeabilizer reagent (102504-100; Seahorse Bioscience). During the assay, 100 μM tetramethyl-p-phenylenediamine (TMPD)/10 mM Ascobate/1 mM ADP (adenosine 5′-diphosphate), and 20 mM azide were injected sequentially. Complex IV-specific activity was calculated by subtracting OCR after addition of TMPD/Ascobate/ADP from OCR after addition of azide.

**Calculation of respiratory control ratio (RCR)**

RCR for oxidative phosphorylation was calculated as previously reported . Briefly, it was assumed that the OCR after the addition of oligomycin represents state 4 respiration and that after FCCP is equivalent to state 3 respiration. After a subtraction of the non-mitochondrial OCR in the presence of antimycin A from all rates, the cellular RCR without (basal RCR) and with the addition of FCCP (maximal RCR) were calculated as the state 3 rate divided by the state 4 rate according to the following equations.

Basal RCR = (basal OCR–OCR after antimycin A) / (OCR after oligomycin–OCR after antimycin A)

Maximal RCR = (OCR after FCCP–OCR after antimycin A) / (OCR after oligomycin–OCR after antimycin A)

**Colony formation assay in soft agar**

The soft agar assay was performed using a kit (ECM570; Millipore, Billerica, CA, USA) according to the manufacturer’s protocol. Briefly, 2500 cells from each condition were plated in a 6-well plate and incubated for 21 days. Colonies with a diameter > 500 μm were counted and representative images were acquired for each condition.

**References**

Cho H, Kim KM, Kim YK (2009). Human proline-rich nuclear receptor coregulatory protein 2 mediates an interaction between mRNA surveillance machinery and decapping complex. *Mol Cell*. **33**, 75-86.

Dixon N, Páli T, Kee TP, Marsh D (2004). Spin-labelled vacuolar-ATPase inhibitors in lipid membranes. *Biochimica et Biophysica Acta (BBA) - Biomembranes*. **1665**, 177-183.

Hill BG, Benavides GA, Lancaster JR, Jr., Ballinger S, Dell'Italia L, Jianhua Z, Darley-Usmar VM (2012). Integration of cellular bioenergetics with mitochondrial quality control and autophagy. *Biol Chem*. **393**, 1485-1512.
